# Phylogeography of rubella virus in Asia: Vaccination and demography shape synchronous outbreaks

**DOI:** 10.1016/j.epidem.2019.100346

**Published:** 2019-09

**Authors:** Brooke A. Bozick, Colin J. Worby, C. Jessica E. Metcalf

**Affiliations:** Department of Ecology and Evolutionary Biology, Princeton University, Princeton, NJ, United States

**Keywords:** Rubella virus, Phylogeography, Metapopulation, Human movement, Epidemic dynamics

## Abstract

Rubella virus causes mild disease in children but for women in the early stages of pregnancy, it can cause spontaneous abortion, congenital rubella syndrome (CRS) and associated birth defects. Despite the availability of an effective vaccine, rubella virus continues to circulate endemically in several regions of the world. This is particularly true in East and Southeast (E/SE) Asia, where control efforts vary widely among countries that are well connected through travel and immigration. It is therefore important to understand how the regional persistence of rubella is affected both by dynamics occurring across countries and susceptibility within countries. Here, we use genetic and epidemiological data from countries in E/SE Asia to explore the phylogeography of rubella virus in this region. Our results underline that metapopulation dynamics are key for rubella persistence and highlight the source-sink population structure of the region. We identify countries that contribute to the regional metapopulation network and link epidemic dynamics to susceptibility profiles within each country. Our results indicate that human movement plays an important role in driving epidemic dynamics in E/SE Asia.

## Highlights

• Metapopulation dynamics contribute to rubella persistence in East/Southeast Asia.
• Circulation patterns differ between rubella viral genotypes 1E and 2B.
• China serves as a regional source population and Japan acts as a regional sink.
• India does not have strong epidemiological links to East/Southeast Asia.
• Taiwan may act as an important sentinel for regional rubella activity.

## 1 Introduction

Rubella virus generally causes a mild childhood infection but can lead to severe birth defects when contracted by women in the early stages of pregnancy. A safe and inexpensive vaccine that confers complete immunity has been available since the 1970s (Plotkin, 2006); it is currently part of the routine immunization program in 159 countries worldwide (World Health Organization, 2017) and has considerably restricted the previously global reach of this pathogen. However, although the virus has been eliminated from the Americas and burdens have been drastically reduced in many developed countries, the WHO still estimates that over 100,000 babies/year are born with congenital rubella syndrome worldwide (Vynnycky et al., 2016; World Health Organization, 2011). Rubella continues to circulate endemically in many countries across both Africa and Asia (Mirambo et al., 2015; Zhu et al., 2015; Pham et al., 2013).

Human movement from endemic countries continues to cause outbreaks of rubella, even in settings where local transmission has been interrupted (Figueiredo et al., 2012; Vauloup-Fellous et al., 2010; Icenogle et al., 2006; Martínez-Torres et al., 2016). As global connectedness grows, it is increasingly apparent that an understanding of how human mobility drives the spread of infectious diseases is necessary to predict and control outbreaks of novel or reintroduced pathogens, especially in a context of growing but incomplete vaccination coverage. The identification of genotypes and/or serologically different viral strains with divergent geographic distributions can be used to distinguish new introductions from ongoing but unobserved circulation (Icenogle et al., 2006; Rivailler et al., 2017). To our knowledge, just a single study has addressed global diffusion of rubella (Cloete, 2014); findings indicated that infection tends to flow out from regions of low vaccination coverage, education index and income rather than necessarily between regions in close geographic proximity. While this result is expected at broad global scales, it is not clear that these patterns will hold at finer spatial resolutions, particularly in regions of Asia where geographically adjacent countries that are strongly connected through travel and immigration have highly variable levels of vaccination coverage.

While countries such as Japan introduced the rubella vaccine to adolescent school girls as early as 1977 (Terada, 2003), populous countries like China and Vietnam only included it in their routine childhood immunization schedule in 2008 and 2015, respectively (Zhu et al., 2012; Gavi TVA, 2019). Theory (Bauch and Earn, 2003) and observation indicate that, for rubella, peaks in incidence occurring annually (Metcalf et al., 2011a; Metcalf et al., 2011b; Metcalf et al., 2013; Wesolowski et al., 2016) or every five years (Terada, 2003; Anderson and May, 1991) are expected in the absence of vaccination, and that erratic transient outbreaks should occur following incomplete introduction of the vaccine (Lessler and Metcalf, 2013; Winter et al., 2018). Data from East and Southeast (E/SE) Asia are incomplete but suggest that a mixture of these patterns is observed (supplementary Fig. 1). From 2011–2014, multiple countries in E/SE Asia, including those reporting high vaccine coverage rates, were affected by a large outbreak, suggesting a degree of regional synchronization. Our understanding of rubella persistence will be strengthened by characterizing the extent to which dynamics within countries are mediated by epidemics occurring in neighboring countries as well as outside the region. Furthermore, the dynamics of susceptibility play an integral role, as outbreaks can only occur where sufficient numbers of susceptible individuals are present. Inferring susceptibility is complex for pathogens with waning immunity, or complex cross-immunity (e.g. influenza), but as rubella infection and vaccination are completely immunizing, estimation of the size of the susceptible population becomes relatively tractable (Bjørnstad et al., 2002) with knowledge on the size of unvaccinated birth cohorts. Characterizing susceptible dynamics will improve our understanding of rubella persistence and may contribute to optimizing public health intervention measures.

Rubella is a single-stranded, positive-sense RNA virus of the *Togaviridae* family. RNA viruses are often amenable to phylogenetic analysis, as their evolutionary dynamics occur on the same time scale as their ecological dynamics (Grenfell et al., 2004). Although only one serotype exists, the CDC has identified two groups of rubella viruses consisting of ten and three genotypes, respectively (World Health Organization, 2013). These genotypes are immunologically identical in terms of both natural infection and vaccination. While the genome comprises approximately 10 kb, genotyping is based on a 739 base pair window of the E1 structural protein, which contains antigenic sites and other important functional domains necessary for cellular invasion (Banatvala and Peckham, 2007). Intraspecific genotypic variation is approximately 5% across genotypes of group 1 and 8% for group 2; maximum variation between groups 1 and 2 is 8–10%. Such genotype classifications are an essential component of virological surveillance and are used primarily to determine whether outbreaks are due to endemic circulation or importation events. Of those genotypes that are currently active, 1E, 1 G, and 2B have achieved the broadest global distributions (Rivailler et al., 2017; Abernathy et al., 2011; Mulders et al., 2016).

In this study, we aimed to assess the importance of metapopulation dynamics to rubella persistence in E/SE Asia. We used publicly available genetic sequences to map pathways of regional spread from 2000 to 2016 and identify countries that act as sources and sinks of viral diffusion. We compared the dynamics of the two most commonly sampled genotypes in Asia, 1E and 2B (Mulders et al., 2016), and found that, despite being immunologically identical, their circulation patterns differ. Our results suggest that there are identifiable sources and sinks for rubella, but that the directionality of spatial spread may be primarily governed by stochasticity and founder effects. We additionally estimated country-specific susceptibility profiles to link temporal trends in rubella incidence and susceptible population size with the timing of viral importation and exportation events. Finally, we discuss limitations arising from analyzing spatially and/or temporally biased samples, a common challenge with studies of this scope.

## 2 Methods

### 2.1 Sequence data

Rubella virus sequences were downloaded from NCBI Genbank in September 2016 (National Center for Biotechnology Information, 2019). We limited our search to sequences from 2000 to 2016 for which a country and date of collection were available. We did not limit our search by demography, so adult, child and CRS cases were all potentially represented in the analysis; not all sequences could be classified demographically due to the limited information available from Genbank. Sequences were aligned using the MUSCLE program in Geneious v5.6.7 (Kearse, 2012), and those that did not map to the 739bp window used for rubella genotype identification (World Health Organization, 2005) were discarded. For the majority of sequences, Genbank provided additional information on either the province or city from which the sample was collected. Separate datasets were created for genotypes 1E and 2B, and each dataset was randomly down-sampled so that no city or province was represented by more than one sequence per year in countries with greater than 50 sequences available (supplementary Table 1). In total, we retained 491 sequences matching our criteria that mapped to the 739bp identification window for genotype 1E and 743 sequences for genotype 2B. In the subsampled datasets, we included 226 sequences from genotype 1E and 250 sequences from genotype 2B. We created and analyzed three versions of each dataset to ensure that our results were robust to differences introduced by the random sampling of sequences (supplementary Table 2). The phylogenies from each of the three subsampled datasets were qualitatively similar for each genotype (supplementary Figs. 2–3).

Sequences collected from within Asia were geo-referenced by country, and sequences from outside Asia were collectively categorized as “Other”. Due to its wide geographic span and cultural ties to countries in Europe, we included sequences from Russia in the “Other” categorization. Sequences from Eurasia and the Middle East were also included in “Other”, as very few sequences were available from these regions.

### 2.2 Phylogenetic analysis

The software package BEAST v1.8.4 (Drummond et al., 2012; Ayres et al., 2012) was used to perform a phylogeographic analysis, from which we obtained the rate and magnitude of viral movement between locations. We used the HKY + I+G substitution model (Hasegawa et al., 1985; Yang, 1994) with a strict molecular clock, the Bayesian skyride coalescent prior (Minin et al., 2008) and an asymmetric, non-reversible, continuous time transition matrix (Ferreira and Suchard, 2008; Gong et al., 2013) for spatial locations using the Bayesian stochastic search variable selection (BSSVS) procedure (Lemey et al., 2009) to identify significant connections. For the BSSVS analysis, a 50% prior probability of identifying no well-supported connections is assumed. The expected numbers of transitions between location states and the timing of these events were calculated using a robust counting procedure (Minin and Suchard, 2008, 2007). For each dataset, three MCMC chains were run for 100 million generations, subsampled every 10,000 steps, and the first 1000 trees were discarded as burn-in. Convergence was assessed using the program Tracer v1.6, to ensure that all estimated parameters had effective sample sizes (ESS) greater than 200. In the case of runs for which ESS values were between 150–200, multiple chains were combined using LogCombiner. Maximum clade credibility trees for each of the sample datasets were produced using TreeAnnotator v1.8.4.

In order to identify well-supported connections between countries, Bayes factors were calculated in the spreaD3 program (Bielejec et al., 2016). As suggested in previous literature (Faria et al., 2013), the following cut-offs were used to classify support for each transition: greater than 100 for strong support, less than 100 but greater than 30 for good support, less than 30 but greater than 10 for support, less than 10 but greater than 3 for weak support and less than 3 for no support.

To identify predictors of viral spread within Asia, we selected one representative dataset for each genotype (sample 1; supplementary Table 2, supplementary Figs. 2–3), removed sequences collected outside E/SE Asia and used a phylogenetic generalized linear model (GLM) as implemented in BEAST v1.8.4 (Lemey et al., 2014). The phylogenetic GLM assesses support for various ecological and sampling factors predicted to influence epidemic diffusion patterns across the phylogeny. We selected 16 different variables encompassing geographic, ecological, demographic, environmental and epidemiological differences between countries to include in the model (see supplementary material). To determine whether the set of variables driving diffusion over the entire 16-year period differed from that responsible for viral spread during the most recent epidemics, we performed the analysis on the dataset of sequences from E/SE Asia collected from 2000 to 2016, as well as the subset of these sequences collected during the height of the regional epidemics, which, according to our dataset, occurred from 2011 to 2015.

### 2.3 Reconstructing the profile of susceptibility

Susceptible populations in countries from which sequences were available were reconstructed using data on vaccination rates and birth rates to predict the size of the unvaccinated birth cohorts (see supplementary material). These were further depleted based on reported incidence using a previously developed framework for modelling natural infection (Takahashi et al., 2015). From this, we estimated the proportion of susceptible individuals within each country; this eases comparison of countries of such different sizes, but is also likely to be epidemiologically relevant as transmission of directly-transmitted childhood infections often scales in a frequency dependent fashion (Bjørnstad et al., 2002). Estimated coverage achieved in rubella vaccination programs, timing and age range of supplementary immunization activities (SIAs; wide age-range campaigns that occur at intervals of 2–4 years), and time-series of rubella incidence are publicly available from the World Health Organization (World Health Organization, 2019). Demographic data on population size, birth cohort size and birth rates are publicly available from the World Bank databank (World Bank, 2019).

## 3 Results

### 3.1 Genotype 1E circulation

**Fig. 1:**
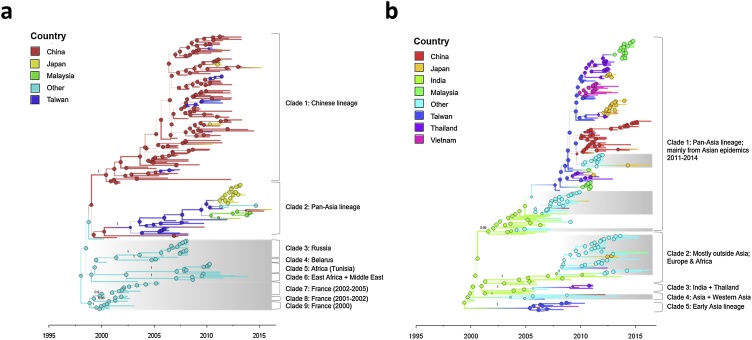
Phylogenetic trees. Phylogenetic trees for a) genotype 1E and b) genotype 2B. Branch colors represent the most probable location state of the lineage at that time. Posterior support values indicated for each of the identified clades. X-axis represented in years. Branch widths sized according to posterior probability, where thicker branches indicate greater posterior support. Clades shaded in grey represent continuous transmission of a lineage outside of Asia. Interior nodes are colored based on their inferred geographic location and sized by the posterior support for this placement, where larger circles indicate greater posterior support.

The most recent common ancestor (MRCA) of all lineages currently circulating in Asia can be traced back to a common ancestor that existed in China in the late 1990s (mean: 1999.02; highest posterior density interval (HPDI): 1997.77–2000.08) (Fig. 1a ). Two major, well-supported lineages containing sequences from Asia are evident: one that became dominant in China (Fig. 1a, clade 1) and one that spread to multiple countries after a period of prolonged circulation in Taiwan (Fig. 1a, clade 2). The remaining clades (Fig. 1a, clades 3–9) are composed exclusively of sequences from outside Asia and are geographically segregated (Russia/Eastern Europe, Africa/Middle East, France). Genotype 1E therefore appears to persist in Asia without continual introductions from outside the region; viral exchange between Asia and the outer world was limited to movement from Asia to other continents.

Clade 1, a large clade of primarily Chinese sequences, corresponds to the previously identified Chinese 1E lineage (Zhu et al., 2015, 2012; Zhu et al., 2016). In line with previous estimates, we identified a divergence time of 2000.4 (HPDI: 1999.53–2001.04). Sequences from Japan, Taiwan, and outside Asia are interspersed throughout the clade but do not cluster together, signifying frequent but sporadic exports from China. The reconstructed ancestral location of four multi-national clusters lies in Taiwan.

Clade 2 contains sequences from multiple countries in and outside of Asia and is characterized by internal clusters with high posterior support (>0.9) that are hierarchically organized by date and country; sequences from similar epidemic periods tend to cluster together and, within these clusters, group by country. This pattern is consistent with intermittent introductions over time. In contrast, finer subdivisions within the Chinese lineage (clade 1) are not well-supported, indicating continuous evolution and persistence of the Chinese 1E lineage throughout China. Clade 2 appears to have descended from an ancestor that originally circulated in China prior to late 2002 before spreading to Taiwan (mean: 2002.73; HPDI: 2001.52–2003.77). The lineage was detected continuously in Taiwan until around mid-2009, when it began to appear elsewhere in Asia.

**Fig. 2:**
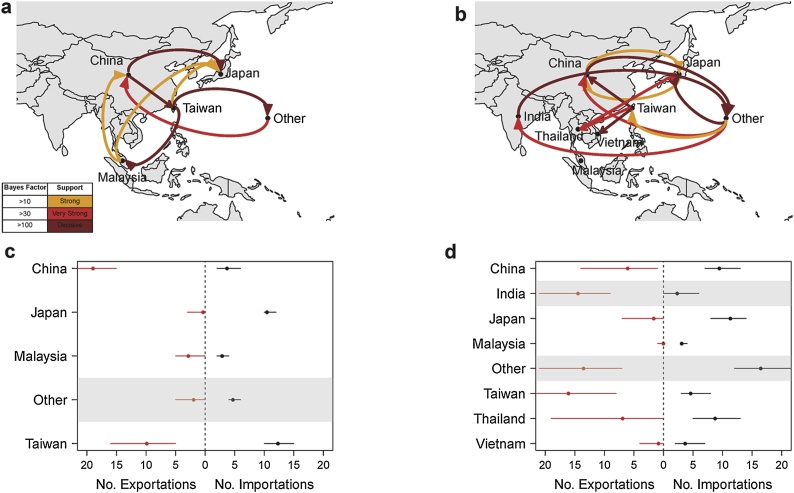
Circulation patterns of rubella virus in Asia. Circulation maps of a) genotype 1E and b) genotype 2B. “Other” represents sequences collected from outside of Asia. Connections between countries are represented by lines colored by their Bayes factor (BF). Dark red (BF > 100) indicates decisive support for the connection, red indicates very strong support (100 > BF > 30), orange indicates strong support (30 > BF > 10). Connections with weak support (10 > BF > 3) are not mapped. Total estimated number of viral importations and exportations per country for c) genotype 1E and d) genotype 2B. Locations identified as being outside the metapopulation circulation network (i.e. no significant connections to the countries of E/SE Asia) are shaded in grey in panels c and d (For interpretation of the references to coloured in this figure legend, the reader is referred to the web version of this article).

Phylogeographic analysis of the circulation patterns of genotype 1E reinforce the importance of China and Taiwan. Within the region, Bayes factors (BF) indicate decisive support (BF > 100) for movement of the virus from China to Japan and Taiwan, and from Taiwan to Malaysia (Fig. 2a ). Total viral exportations from China far exceeded importations (Fig. 2c). Numerous imports and exports in and out of Taiwan are tied to the country’s significant connections to both East and Southeast Asia (supplementary Fig. 13). Additional analyses of datasets containing only sequences collected from recent cases in Taiwan (occurring from 2010 to 2015) confirmed that older sequences from Taiwan were not biasing the ancestral state characterization (see supplementary information). In contrast, there was no support for viral migration out of Japan, where importations far exceeded expected exportations (Fig. 2c, supplementary Fig. 11). Of the 16 predictors tested in the phylogenetic GLM, there was weak support for geographic distance metrics and destination country sample size having an effect on viral diffusion when examining the entire time period (supplementary Fig. 6). However, none of our selected predictors were significant when considering only sequences collected during the recent epidemics from 2011 to 2015 (supplementary Fig. 7).

### 3.2 Genotype 2B circulation

The common ancestor of all sampled global lineages existed in India in the late 1990s (mean: 1999.3; HPDI 1998.33–2000.15) (Fig. 1b). However, this could be biased if few locations were sampled during this period; according to the World Health Organization, reporting of rubella cases in many countries did not start until 2005 (supplementary Fig. 1). Genotype 2B circulates widely throughout the region, as evidenced by the large multi-national clade containing many E/SE Asian sequences from the 2011–2014 epidemics (Fig. 1b, clade 1). Similar to the pan-Asia lineage of genotype 1E, sequences cluster by outbreak and country. Correspondingly, there is no evidence for long-term persistence of country-specific lineages. The existence of a distantly-related Asian clade predating the 2011–2014 epidemics (Fig. 1b, clade 5), and the probable placement outside of Asia of the ancestor of all E/SE Asian sequences in clade 1 (posterior: 0.65) suggests that the recent outbreaks were caused by a lineage newly introduced from outside the region, rather than by an E/SE Asian lineage that persisted across inter-epidemic periods. In total, we identified five well-supported clades, all of which contain sequences from within and outside of Asia. Further, there is strong support for the progenitors of each of these clades to have existed outside of E/SE Asia, indicating that historic circulation of genotype 2B was maintained by introductions from outside the region.

Few direct transitions between India and E/SE Asia are observed, suggesting that viral exchange is minimal; with the exception of the early Asia lineage (clade 5), all groups of E/SE Asian sequences evolved from an ancestor predicted to have existed outside of Asia. However, sparse sampling from more recent outbreaks in India could potentially bias this conclusion as the majority of sequences from India were sampled from 2004-2008. As a populous country with low vaccination coverage (Dewan and Gupta, 2012), the existence of a sustained epidemic trough post-2008 is unlikely. However, the presence of two Indian sequences from 2011 and 2013 which cluster with others from outside of E/SE Asia supports the conclusion of limited direct viral exchange.

Phylogeographic analysis of the circulation patterns of genotype 2B again suggests an important regional role for Taiwan (Fig. 2b, supplementary Fig. 13). We find decisive support (BF > 100) for movement from Taiwan to both China and Southeast Asia. Additional analyses of datasets containing only sequences from recent cases in Taiwan (occurring from 2010 to 2015) show that exportations from Taiwan scale with the number of recent sequences included in the analysis, suggesting high genetic diversity among recently sampled Taiwan sequences rather than a collection of highly homogenous sequences sampled from a few related outbreaks (see supplementary material). Bidirectional exchange between China and Japan is also strongly supported (BF > 10) although few movements between these countries occurred. Again, far more importations than exportations are observed for Japan (Fig. 2d, supplementary Fig. 11), and significant movement from Thailand to Japan is apparent (Fig. 2b, supplementary Fig. 14). Viral exchange between India and countries outside of Asia is strongly supported (BF > 100), though we find no significant connections between India and E/SE Asia, suggesting that India does not play a role in the E/SE Asia metapopulation. None of the 16 predictors included in the phylogenetic GLM were significantly correlated with viral diffusion when considering the entire period from 2000 to 2016 (supplementary Fig. 8). When considering only sequences collected during the recent epidemics from 2011 to 2015, geographic distance and destination country sample size were marginally significant (supplementary Fig. 9).

### 3.3 Viral demography in the context of epidemiological dynamics

WHO case data indicates that incidence increased in most of the focal countries between 2010 and 2015 (supplementary Fig. 1a). With the exception of Vietnam, all focal countries report coverage of rubella-containing vaccine (RCV1) to be above 90% by 2012 (supplementary Fig. 1b), suggesting a level of susceptibility well below the theoretical threshold necessary for elimination based on previous estimates of R_o_ ranging from 3 to 8 (Metcalf et al., 2011b) and a vaccination coverage threshold of 80% (1-1/R_0_ for R_0_˜5). That rubella continues to circulate in many of these countries suggests uncertainty in reporting of coverage (Lessler et al., 2011) and/or heterogeneity underlying national coverage rates (Takahashi et al., 2017).

For each of the focal countries, accumulations of susceptible individual precede each peak in reported cases (supplementary Fig. 1c). Apart from Thailand and Taiwan, heightened susceptibility between 2009 and 2014 was apparent. Although total proportion susceptible in Taiwan was consistently low, estimated accumulations of susceptible individuals occur around 1999 and 2006. In Thailand, the estimated proportion susceptible remained stable and relatively low throughout the entire period.

Rubella importation can only be successful if sufficient susceptible individuals are present. The timing of viral importation events into each country appear to precede epidemic peaks (supplementary Fig. 4) and tend to occur during local peaks in susceptible population size (supplementary Fig. 5). For example, imports of genotype 2B into China are observed around 2006 and 2009, the times during which susceptible population size peaked just prior to each major outbreak. As the epidemiological data suggests that the susceptible population size in China falls after 2005 (supplementary Fig. 1c), we might expect that there are fewer opportunities for the introduction of new strains. However, if erratic local dynamics cause extinctions in areas where not enough susceptibles are present, susceptibles throughout China will eventually accumulate enough to allow new introductions. In support, the phylogeny suggests that introductions continued to happen in China across the time course of this study. Further, the small spike in introductions to China in 2009 coincides with an accumulation of susceptibles following the epidemic in 2008 even though the overall proportion susceptible is consistently decreasing (supplementary Fig. 5). As only 1–2 epidemics occurred during the time period of study in each of the focal countries, we were unable to statistically test this observation, but in the context of the previously described phylogenetic patterns it is suggestive of the importance of metapopulation processes in the epidemic dynamics of rubella in this region.

**Fig. 3:**
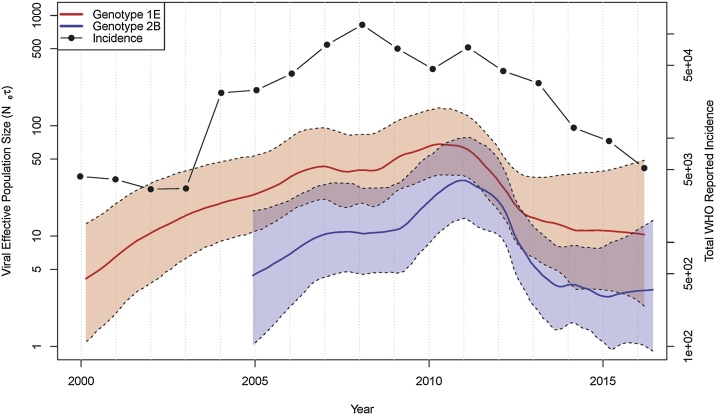
Viral effective population sizes (N_e_). Estimates of viral N_e_ in E/SE Asia for each genotype plotted with total reported cases across the six focal countries. Sparse sample collection precluded estimating dynamics for genotype 2B prior to 2005.

Demographic reconstruction of the viral effective population sizes (N_e_) of each genotype within E/SE Asia reveals strikingly similar patterns: two periods of epidemic growth interspersed with an approximately two-year period of stasis (Fig. 3). Peaks are apparent for both genotypes around 2011, whereas growth phases are observed from 2005 to 2007 and from 2009-2011. While improved sampling could underlie this sharp increase, it also coincides with the period in which several Asian countries experienced rubella epidemics (supplementary Fig. 1a). Broad concordance between peaks in total incidence and N_e_ are observed, particularly for genotype 2B (Fig. 3).

## 4 Discussion

To understand whether metapopulation dynamics contribute to the persistence of rubella across neighboring countries that vary in their level of vaccination coverage, we combined both phylogenetic and epidemiological lines of evidence. Our analysis indicates that circulation patterns may differ between rubella virus genotypes 1E and 2B. As no evidence of a substantial competitive advantage for either subtype exists to date, these differences therefore suggest that the current distributions are likely a product of stochastic forces and founder effects stemming from the timing of their initial invasion. We confirmed the presence of a major lineage of genotype 1E evolving in China and found evidence for an additional 1E lineage circulating throughout Asia. Although some exchange with countries outside of the region was apparent, recent genotype 1E circulation in Asia appears isolated from the rest of the world. In contrast, we found evidence for multiple, recent introductions of genotype 2B from outside of Asia, suggesting continual global exchange. There was a clear increase in the effective population size of each genotype just prior to the 2011–2014 epidemics in Asia, suggesting that simultaneous outbreaks of both occurred. While China serves as a regional source population, particularly for genotype 1E, Taiwan likely acts as an important indicator for regional rubella activity across East and Southeast Asia. In line with high reported levels of vaccination coverage, we find that Japan is a regional sink. Further, India does not have strong epidemiological links to E/SE Asia.

While other studies have looked at global rubella circulation, our study focuses on regional spread in an area of endemic transmission. Our results suggest that metapopulation dynamics are integral to the persistence of the virus, as lineages tend to circulate continuously across the region without a strong signal of spatial isolation within countries between epidemics. Although clear source and sink populations were evident for genotype 1E and, to some extent, genotype 2B, we also identified widely distributed lineages that were introduced from outside Asia. However, we were not able to identify any strong predictors of viral diffusion throughout the region for either of the genotypes we studied. Although geographic proximity appeared to be weakly correlated with viral diffusion, this relationship was erratic among the two genotypes. This finding was likely spurious as distance is probably a poor measure for the connections that are relevant to rubella spread. Our finding that viral persistence is maintained through a complex, lineage-specific circulation network is similar to that described for other childhood diseases in the E/SE Asia region, notably enterovirus A71 (Thao et al., 2017). Limited viral exchange occurred between India and E/SE Asia over the period of our study, an observation likely due to cultural differences across the Asian continent. Owing to historical events, India is more strongly connected through travel and immigration with countries in Europe, North America and, more recently, the Middle East (Naujoks, 2009; United Nations, 2016). As very few sequences are available from the Middle East and other countries in Western Asia and the Pacific where large populations of Indian immigrants reside, we cannot say at this time whether these connections are relevant to the spread of rubella. It presents an interesting avenue for future research, in particular because of observed differences in epidemic dynamics between India and the Asia-Pacific region for other infectious diseases (Pons-salort et al., 2015). Characterizing patterns such as the limited relevance of imports from India for re-introduction of rubella in E/SE Asia will become increasingly important as the region approaches rubella elimination.

Given the introduction of rubella-containing vaccine to all children in Japan in 1989, Japan’s role as a sink for viral importation is unsurprising. The outbreaks represented in this study occurred primarily among adult men that were not vaccinated during childhood (Tanaka-Taya et al., 2013), as initial vaccination campaigns in Japan starting in 1977 only targeted adolescent schoolgirls (Terada, 2003). Though outbreaks in Japan continue to occur, the introduction of the MMR vaccine in 1989 and the policy switch towards the targeting of infants and young children has resulted in epidemics of decreased magnitude (Terada, 2003) with an increased average age of infection (Sugishita et al., 2015; Kinoshita and Nishiura, 2016). This, coupled with an increasing share of foreign residents immigrating from Southeast Asia (Green, 2017), make Japan a prime location for the importation of viral lineages, an observation which, paradoxically, is a metric indicating successful control. It remains to be seen how rubella dynamics throughout the region will affect Japan as it continues to near elimination.

While China’s importance is easily explained by its large population size, relatively recent introduction of routine rubella vaccination, and prevalence of travel connections, our finding of Taiwan’s role as a significant disseminator of infection (also suggested previously (Cloete, 2014)) is less obvious. Rubella is a notifiable disease in Taiwan and a subset of sequences collected from 2005 to 2013 have been well characterized epidemiologically. Many of these sequences were traced to importation events from Vietnam, and to a lesser extent Malaysia, Thailand and China, while other outbreaks that were untraceable in origin occurred within immigrant communities (Cheng et al., 2013). Although our phylogenetic analysis identifies Taiwan as an ancestral location, an alternative explanation is that the high intensity of surveillance could lead to outbreaks being identified earlier here than in the countries from which they originated. Under this hypothesis and coupled with reported vaccination rates of greater than 90% since 2000, Taiwan is likely not an actual source of infection; instead, Taiwan probably acts as a sentinel for the rest of the region.

Reinforcing this, in addition to its well-established surveillance system which facilitates viral detection and characterization, there are multiple factors that predispose Taiwan to rubella importations. Past serological studies show that seronegativity is higher among immigrant women (Lin et al., 2010; Wang et al., 2007) and until 2002, Taiwan did not require proof of rubella vaccination upon immigration (Lin et al., 2010; Taiwan Centers for Disease Control, 2019). Further, an increasing proportion of immigrants now come from nations in Southeast Asia, where endemic rubella transmission is still widespread and vaccination has only recently been introduced (Lin et al., 2010; Wang et al., 2007; Tsay, 2015). In addition, from 2006 to 2010, 15–28% of Taiwanese men were married to foreign-born women, primarily from Southeast Asia (Lin et al., 2010). Finally, Taiwan has recently experienced an increase in international air travel. In 2014, 9.9 and 11.8 million people, respectively, travelled in to and out of the country and an estimated 84–88% of these travelers either visited or visited from countries in E/SE Asia (Shih et al., 2016). While these patterns are unlikely to outweigh high vaccination coverage and result in Taiwan contributing importantly to regional dynamics, such high rates of immigration and international travel, coupled with its strong geopolitical ties to China, a clear source of rubella infection, makes Taiwan an ideal sentinel for the identification of new lineages in the region.

The observed simultaneous increases in the effective population size of both genotypes is striking given that rubella virus immunity is completely cross-protective. Previous work has shown that the interpretation of viral effective population size estimates drawn from Bayesian coalescent skyline techniques should focus on changes over time, as observed increases in N_e_ correlate best with increases in incidence at the start of an epidemic (Frost and Volz, 2010). The synchronized phases of exponential viral population growth (2005–2007 and 2009–2011) that coincide with the two epidemic periods observed in the WHO case reports are evidence that both genotypes simultaneously circulated and contributed to epidemics in the region. This is surprising for a fully cross-immunizing infection, as it suggests that the entire E/SE Asia region is nearly in phase. While we would not expect two consecutive years of viral population growth during any single rubella epidemic in a given location, closely synchronized outbreaks across multiple countries could appear as consecutive years of growth when taken together. Two scenarios may be invoked as an explanation for epidemic synchronization across the region.

First, a country or city might act as a major pulse driver, akin to that of London for pre-vaccination measles epidemics in the United Kingdom (Grenfell et al., 2001). However, this scenario would require that only one major location play a key role and that sink populations are only weakly connected such that rescue effects after epidemic extinction are rare. These conditions are unlikely, given that many cities are above the critical community size for maintaining persistent rubella circulation (Metcalf et al., 2013) and that high international connectivity in E/SE Asia has been cited as a possible explanation for the persistence of other viruses (Russell et al., 2008). However, we find strong evidence that China functions as a source population for genotype 1E. Given that we also did not find support for repeated introductions from outside Asia for this genotype, there is a possibility that epidemiological dynamics in China drive the regional spread of genotype 1E.

The second scenario we envision to explain synchronized phases of viral growth emerges from extremely high regional connectivity. If the existence of a connection, rather than the magnitude of movement across that connection, is the chief predictor of outbreak timing, we might expect that an epidemic arising in one location will immediately ignite outbreaks in all connected populations whose susceptible population size is above the threshold necessary for a sustained epidemic to occur. Comparable seasonal travel patterns across settings (e.g. driven by similar timing of school holidays (Wesolowski et al., 2017)) will reinforce this alignment of timing, creating a positive feedback loop to further synchronize dynamics. Sporadic introductions from weakly connected locations that are not part of the core circulation network would then reseed epidemics once the pool of susceptibles has again reached an appropriate size. This scenario is consistent with our study, in that (1) the magnitude of connectivity measured by travel flows was not predictive of viral diffusion, (2) extensive travel and immigration ties exist between countries throughout the region and (3) repeated introductions from outside E/SE Asia were observed for genotype 2B.

An inherent limitation of using publicly available data is the inevitability of skewed and potentially biased sampling. Sequences sourced from Genbank originate from multiple studies with different methodologies and different motivations for collection. As a result, the quantity of data publicly available from any single location or time period varies. The number of available sequences from Japan, Taiwan and China, where surveillance is more extensive, far exceeds that from countries in Southeast and South Asia, where rubella is often not a notifiable disease. Further, even in the most well sampled locations, sampling biases exist over both space and time. Despite our efforts to correct for this, we cannot exclude the possibility that additional, unsampled lineages circulate, or have circulated, in Southeast Asia and India where sampling is sparse. Further, our analysis does not account for viral exchange with countries from which no samples are present. The possibility therefore remains that other countries may also act as sources. Broader sampling throughout Asia, both in terms of locations represented and the quantity of sequences available, as well across the globe, particularly from rubella-endemic countries that lack access to vaccination, will further refine our understanding of rubella circulation and persistence. Literature on the historical genotypic characterization of global rubella viruses indicates that many subtypes and lineages have gone extinct from 1960 through to the present (World Health Organization, 2013) and it remains to be seen whether, for example, the introduction of genotype 2B into China will lead to local extinction of genotype 1E. However, as sampling is not consistent or complete across this region, it is difficult to say with any certainty whether lineages have actually gone extinct, are still present at low levels, or are circulating in countries with minimal available data. Our results suggest that rubella lineages are not confined to specific countries and therefore that surveillance and elimination efforts must take into account the epidemiology of the region as a whole and population movements between countries in order to be successful.

## Ethics

All genetic data were publicly available and anonymized; thus, this study did not require approval by an ethics committee or institutional review board.

## Data, code and materials

Genbank accession numbers for all sequences used in this study can be found in supplementary table 2.

## Competing interests

The authors declare no competing interests.

## Authors’ contributions

B.A.B and C.J.E.M. conceived the analysis. B.A.B., C.J.E.M and C.J.W. designed the analysis. B.A.B. performed the analysis. B.A.B., C.J.E.M. and C.J.W. wrote the paper.

## Funding

This work was supported by a grant from the Bill and Melinda Gates Foundation (CJEM, BAB; OPP1094793).
