# Supplementary material for: Phylogeography of rubella virus in Asia: Vaccination and demography shape synchronous outbreaks
Source: Epidemics. 2019 Sep;28:100346. doi: 10.1016/j.epidem.2019.100346 (PMC6731519; doi:10.1016/j.epidem.2019.100346)
Supplement: Supplementary file 1 [file mmc1.docx]

**Supplementary material**

**Methods**

***Reconstructing the profile of susceptibility***

To reconstruct the pattern of susceptibility, we initially estimated the size of the pool of unvaccinated individuals. Focusing first on the routine program, every year, approximately (1-*v_t_*)*B_t_* unvaccinated children enter the population, where *B_t_* is the size of the birth cohort in year *t*, and *v_t_* is vaccination coverage achieved within the routine program in that year. We assumed that in years prior to the introduction of rubella-containing vaccine (RCV) into the routine program, vaccination coverage was negligible. Since immunity from vaccination is life-long, the cohort of one-year old children vaccinated in year *t* will retain this coverage as two-year olds in year *t*+1; thus, we mapped coverage from routine vaccination across cohorts. To add vaccination resulting from Supplementary Immunizing Activities (SIAs), we additionally depleted these cohorts, according to coverage achieved within the appropriate years, by targeting the appropriate age ranges. Since evidence for the scope of SIAs suggests that it is limited, we assumed that the first and any subsequent doses are overlapping (i.e., reach broadly the same children) and reflected this by assuming a maximum coverage of SIAs among remaining susceptibles of 70%.

The data on routine and SIA coverage was then combined with estimates of age-specific infection to estimate the fraction of the population susceptible over the time course. To account for the impact of natural infection on susceptibility, we assumed that individuals acquire rubella according to a constant hazard of infection of age. This was set such that in 1980, 90% of individuals were infected by age 20, as *P*(susceptible at age *a*)=*exp*(-0.2*a*). To capture variation in the hazard of infection over time, we reduced this rate according to the ratio of cases reported relative to those in 1980, yielding (infection by age ??) = 1 − (-0.2??r), where *r* is assumed to be 1 prior to the start of the reported incidence data, and, subsequent to this, is estimated by using a smooth-spline fit to account for missing years. This is clearly a simplification given the potential for erratic outbreaks, but as we were uncertain of the ability of the existing data to inform such spatial and temporal heterogeneity, we chose to use this relatively simple one-parameter approach.

***Phylogenetic generalized linear model predictors***

The following predictors of rubella virus diffusion were considered in our phylogenetic generalized linear model. With the exception of the geographic distance metrics and passenger flux, which provide measures of similarity/difference between locations, all predictors were calculated and tested with regard to both the origin and destination country.

• **Geographic distance metrics**

Geographic distance was calculated as the great circle distance between country centroids. We additionally considered the mean great circle distance between all pairs of airports with flight connections from two countries, as well as the minimum distance across all airport pairs from two countries.

• **Latitude**

To test whether broad scale climate differences play a role in rubella diffusion, we included the absolute latitude of the centroid of each country.

• **Connectivity through air travel**

As a measure of connectivity, we used estimates of the total annual number of passengers traveling between countries through international air travel as predicted by ^1^. We also tested the total number of routes flown between two countries using data downloaded from OpenFlights.org.

• **Demographic metrics**

Population size and density estimates were downloaded from the World Bank data repository (data.worldbank.org). As rubella primarily infects young children, we additionally tested country-specific estimates of birth rate, total births and population size of children under 14 years of age based on data from the United Nation’s World Population Prospects 2015. Demographic data for Taiwan was obtained from The Statistical Yearbook of the Republic of China (eng.dgbas.gov.tw). We used the population size of children aged 0-14 to match the resolution of the available data for Taiwan.

• **Human development index**

We used the 2013 Human Development Index (hdr.undp.org) as a measure of economic and developmental disparity between countries. The Human Development Index takes into account indicators such as life expectancy, expected years of schooling and gross national income per capita to present a broad overview of the level of development of a country.

• **Vaccine availability**

WHO/UNICEF vaccination coverage estimates by year were available from http://www.who.int/immunization/monitoring_surveillance/routine/coverage/en/index4.html, and the year of introduction of rubella containing vaccine into a country was obtained from http://www.who.int/immunization/monitoring_surveillance/data/en/. To test the effects of vaccination at different time points, we considered mean coverage for the entire time period (2000-2016), mean coverage for the entire time period and five years prior (1995-2016), coverage in 2008, and coverage in 2013.

• **Sample size**

Sample size was calculated as the total number of sequences included in the analysis from each individual country.

**Analyses**

***Investigating the role of Taiwan in the E/SE Asia metapopulation***

To determine to determine the extent to which temporal bias in samples obtained from Taiwan influenced the ancestral state reconstruction and source/sink analysis, analyses were rerun after the removal of older Taiwan sequences. Based on natural breaks in the data, “older” Taiwan sequences were defined as those collected prior to 2010 (supplementary figure S16). We removed all sequences from Taiwan collected prior to 2010 from each of the three sample datasets for each subtype and replaced them with sequences collected from 2010 (inclusive) onwards. For genotype 1E, this was straightforward: due to subsampling, 26 Taiwan sequences had been included in the original analysis, and there were 21 sequences available post-2010 that were inserted into the alignment as a replacement. For genotype 2B, 41 sequences were available post-2010 from Taiwan, whereas only 21 total Taiwan sequences had been included in the original analysis. We investigated both (1) replacing the set of Taiwan sequences with all 41 sequences collected post-2010 and (2) replacement with a sampled subset of 24 sequences collected post-2010 from Taiwan. Our subsampling methodology was similar to that described in the main text; here we chose no more than 3 sequences per city per year to best recapitulate the original number of Taiwan sequences included. Since the results in the initial analysis had been consistent across the 3 sample datasets, we tested 3 different Taiwan sequence subsets inserted in the one originally sampled dataset that was used to create the figures and maps presented in the main results section of the paper.

For genotype 1E, removing older Taiwan sequences reduced the estimated mean number of exports from 10 (BCI 5-15) to 5 (BCI 0-10), and had only a minor effect on the number of imports, increasing it slightly from 12 (BCI 10-15) to 14 (BCI 10-16) (supplementary figure S17 (left)).

For genotype 2B, removing older Taiwan sequences and replacing them with a subset of recent Taiwan sequences slightly decreased the estimated mean number of exports from 15 (BCI 6-20) to 14 (BCI 5-20) and didn’t affect the number of imports (figures S17 (center)). The mean and confidence interval remained the same. However, the mean number of exportations from China increased from 5 (BCI 1-15) to 13 (BCI 2-20). In contrast, replacing with the entire set of 41 new sequences drastically increased the estimated mean number of exports from 15 (BCI 6-20) to 30 (15-40), but did not affect the number of imports (figure S17 (right)). The scaling of exportations with the number of sequences suggests high genetic diversity among recently sampled genotype 2B sequences from Taiwan, rather than a collection of highly homogenous sequences sampled from a few related outbreaks. These results are most consistent with a country that is nearing elimination, for which most outbreaks stem from isolated importations that infect populations that are under-vaccinated (i.e. immigrant communities in this case). The molecular epidemiology backs up these conclusions. From the available data, we cannot draw a definitive conclusion as to why Taiwan appears as a source, but hypothesize that heightened surveillance in this country allows even small outbreaks to be detected, and that detection occurs at a much faster rate than in the countries from where the importation occurred.

In a comparison of phylogenies constructed using all sequences and with Taiwan pre-2010 sequences removed, some changes to the internal structure of the trees, but the qualitative results remain the same. Removing older Taiwan sequences from the Genotype 1E tree changes the relationship of the pan-Asia clade to be more closely related to the lineage circulating outside of Asia than to the endemic Chinese lineage (figure S18). However, the endemic Chinese lineage is still recovered, and the pan-Asia lineage is still derived from an ancestor that circulated outside of Asia. An ancestor circulating first in China and then in Taiwan is still predicted to be ancestral to the pan-Asia clade, despite the removal of the older Taiwan sequences.

The structure of the Genotype 2B tree remains largely unchanged when older Taiwan sequences are removed and all 41 post-2010 sequences are included in the analysis (figure S19). Sequences from the recent epidemic still form their own clade, and sequences from in and outside of Asia are still interspersed throughout the tree. In addition, the phylogeographic analysis still place Taiwan as the backbone of the 2010-2015 outbreak. The older Taiwan sequences (that were removed) largely clustered at the base of the original tree and were from a previous epidemic lineage; they were not closely related to the lineage from outside Asia that gave rise to the later outbreak.

Differences between the phylogenies of the 3 subsets of Taiwan sequences are noticeable, particularly in the predicted country representing the backbone of the 2010-2015 epidemics (figure S20). In 2 of 3 samples, Taiwan remains ancestral to the 2010-2015 outbreaks, however in subset 3, an ancestor circulating in China is predicted to be ancestral to the later outbreaks across E/SE Asia. While posterior support is high for clades of sequences collected from a single country, posterior support weakens further back in time. Difficulties resolving tree structure could contribute to uncertainty regarding ancestral source populations.

**References**

1. Mao, L., Wu, X., Huang, Z. & Tatem, A. J. Modeling monthly flows of global air travel passengers: An open-access data resource. *J. Transp. Geogr.* **48,** 52–60 (2015).

**Tables**

|  | Genotype 1E | | Genotype 2B | |
| --- | --- | --- | --- | --- |
| Country | *Subsampled*  *(All available)* | *2011-2015 Epidemics* | *Subsampled*  *(All available)* | *2011-2015 Epidemics* |
| China | 101 (337) | 40 | 32 (216) | 28 |
| Japan | 28 | 26 | 33 (204) | 31 |
| India | - | - | 29 | - |
| Malaysia | 5 | 4 | 29 | 29 |
| Taiwan | 26 (56) | 7 | 21 (63) | 7 |
| Thailand | - | - | 23 | 22 |
| Vietnam | - | - | 14 (51) | 12 |
| Outside Asia | 65 | - | 74 | - |

**Table S1. Dataset Information.** Number of sequences per dataset. “All available” indicates the number of sequences downloaded from Genbank and “Subsampled” refers to the number of sequences remaining after downsampling by collection year and location. Phylogenetic GLM analyses were performed on one subsampled dataset using sequences from countries within E/SE Asia (i.e. removing sequences from India and outside Asia) as well as on a further pruned dataset consisting only of sequences from E/SE Asia collected from 2011-2015 (“2011-2015 Epidemics”).

**Table S2. Sequence Information.** Genbank accession number, collection country, collection year, genotype and sample dataset(s) that the sequence was included in for all rubella sequences used in this study.

**Figures**

**
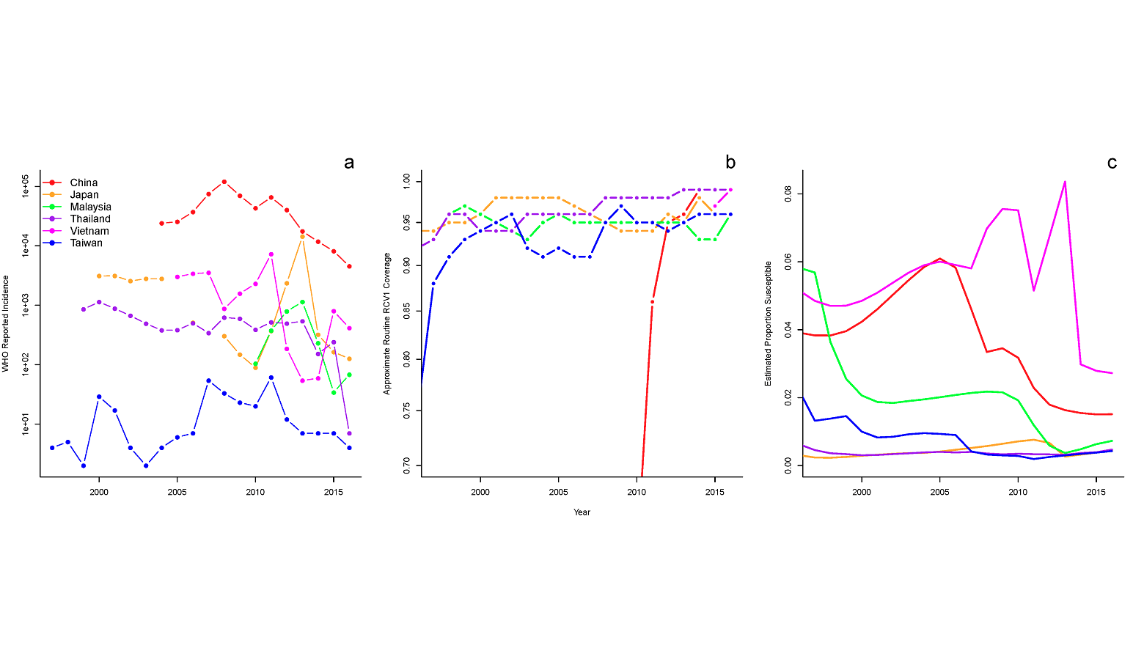
**

**Fig. S1. Epidemiology of rubella virus.** a) Rubella incidence reported by the WHO (log scale); b) coverage of the first dose of rubella-containing vaccine, reported by the WHO; c) inferred proportion susceptible


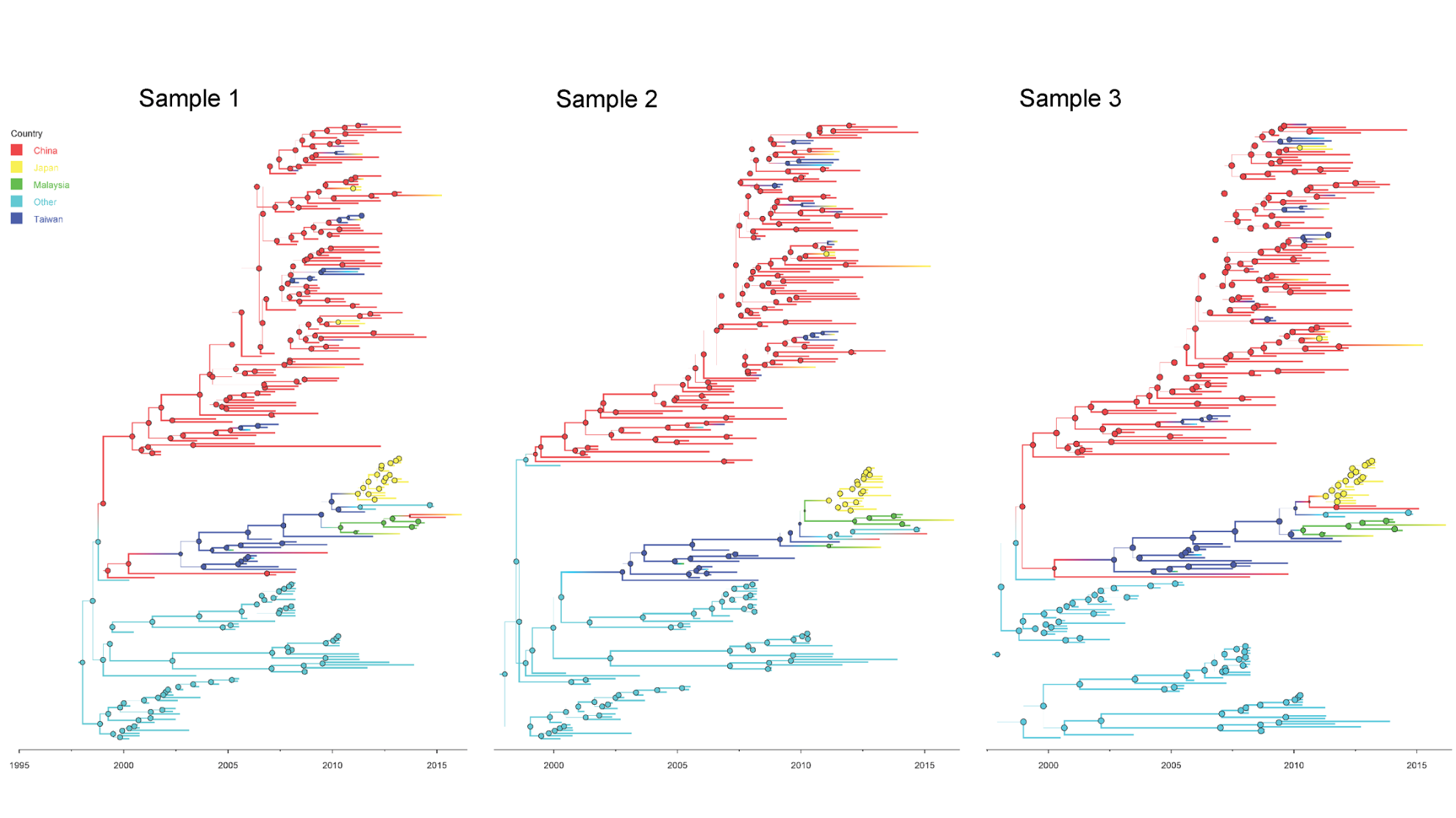


**Fig. S2. Genotype 1E phylogenies for each of the three subsampled datasets.** Branch colors represent the most probable location state of the lineage at that time and node size represents posterior support for the location state of each node. Branch widths sized according to clade posterior probability.


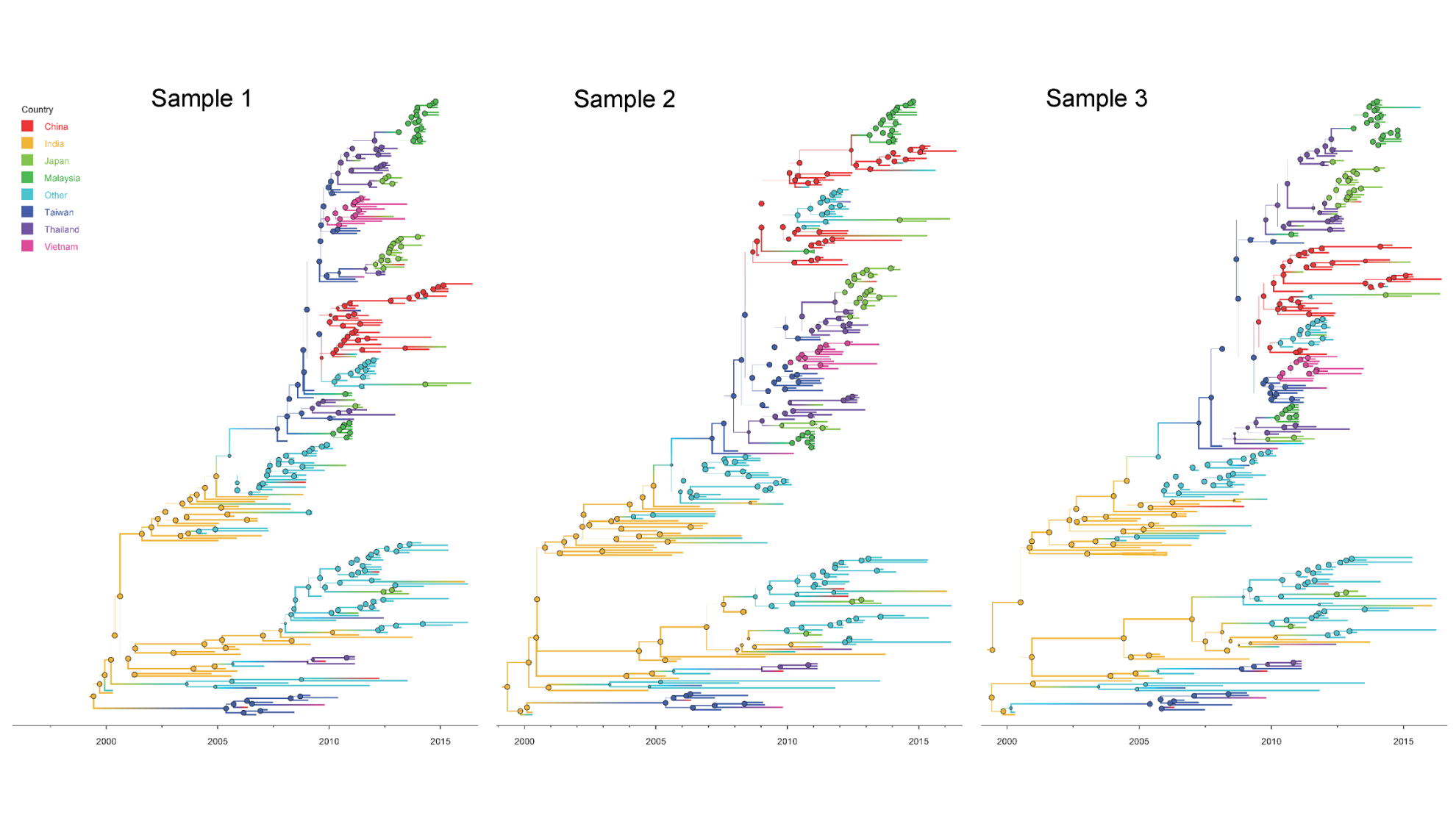


**Fig. S3.** **Genotype 2B phylogenies for each of the three subsampled datasets.** Branch colors represent the most probable location state of the lineage at that time and node size represents posterior support for the location state of each node. Branch widths sized according to clade posterior probability.


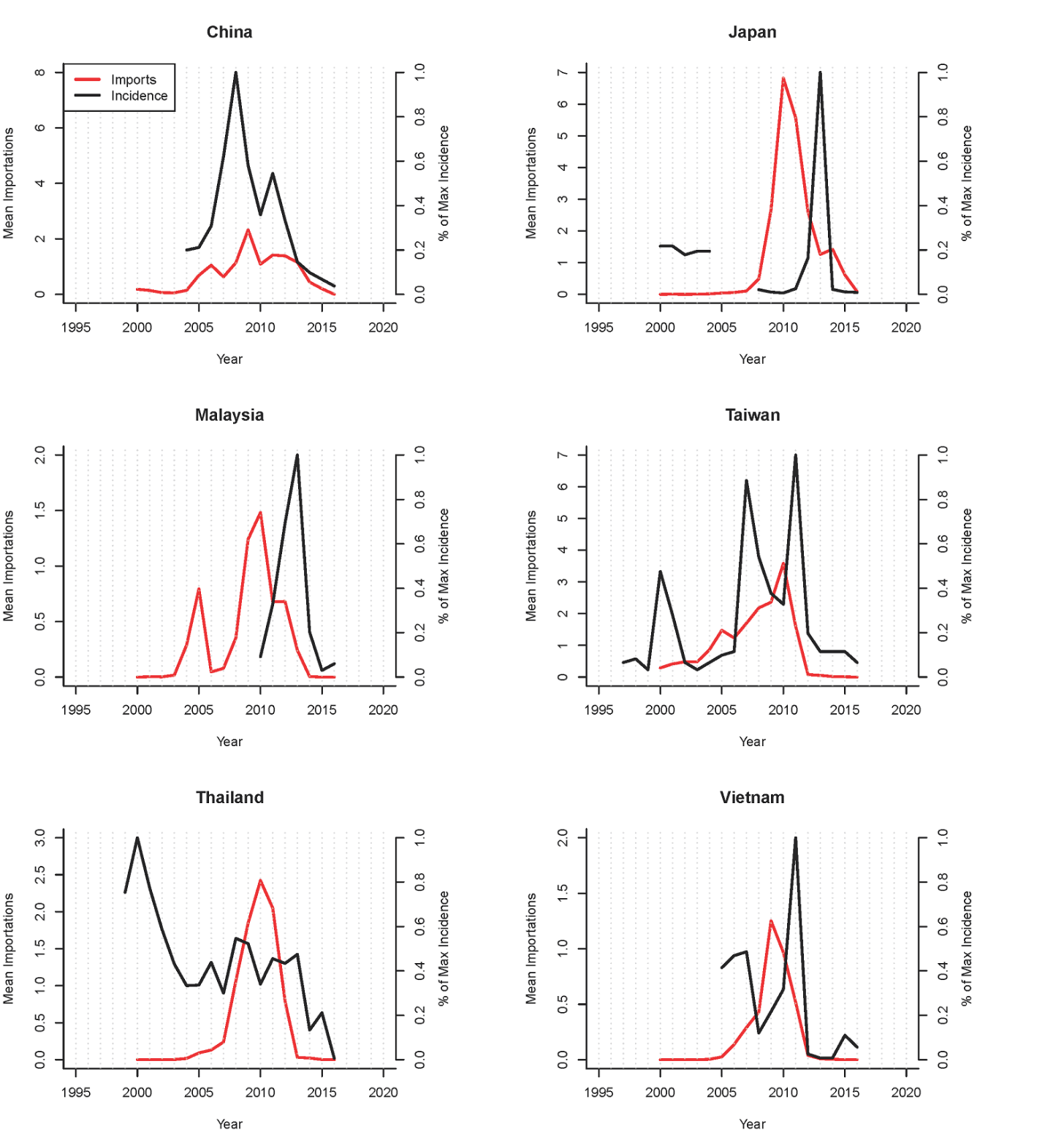


**Fig. S4. Country specific estimates of importation events from genetic data superimposed on WHO incidence estimates.** To highlight trends in peak case counts, incidence is displayed as proportion of maximum incidence recorded from 2000-2016.

**
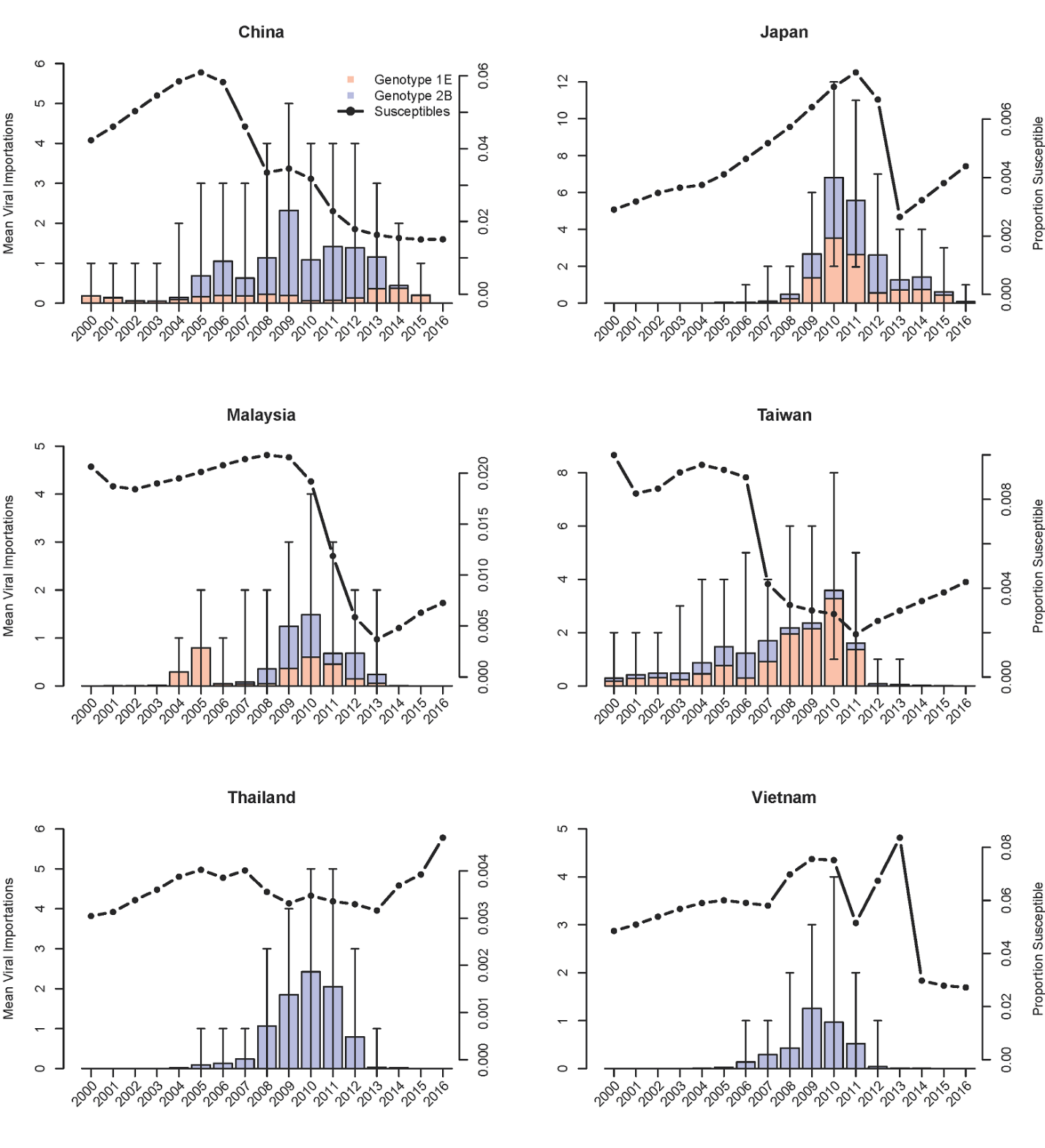
**

**Fig. S5. Importation events and susceptibility profiles.** Stacked bars represent the mean number of importations of genotype 1E (red) and genotype 2B (blue) per year. Error bars represent 95% Bayesian credible intervals. Superimposed is the estimated proportion of susceptible individuals in each country.


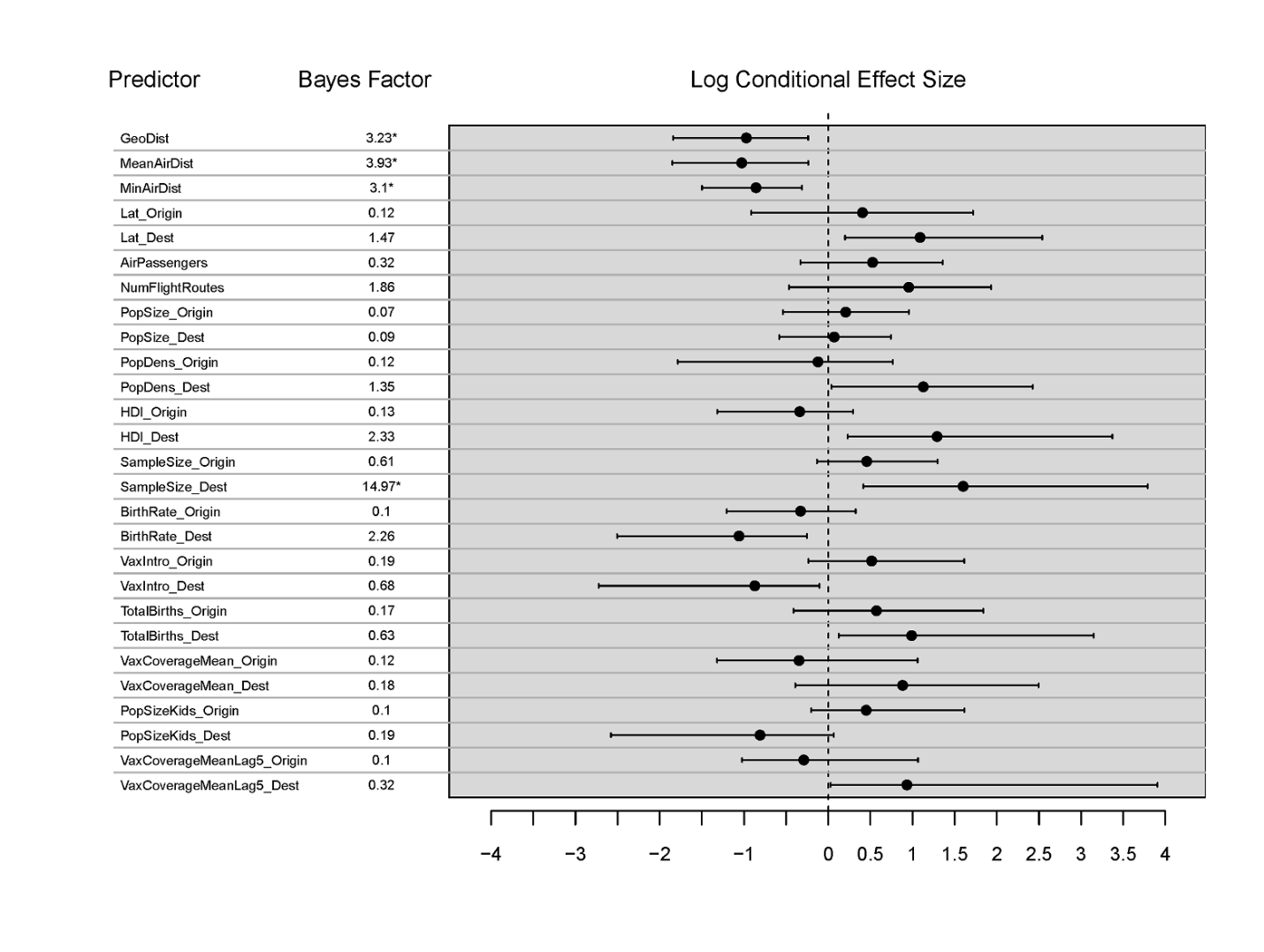


**Fig. S6. Bayesian phylogenetic GLM analysis of all E/SE Asia sequences for genotype 1E (2000-2016).** An asterisk follows Bayes factors for significant predictors.


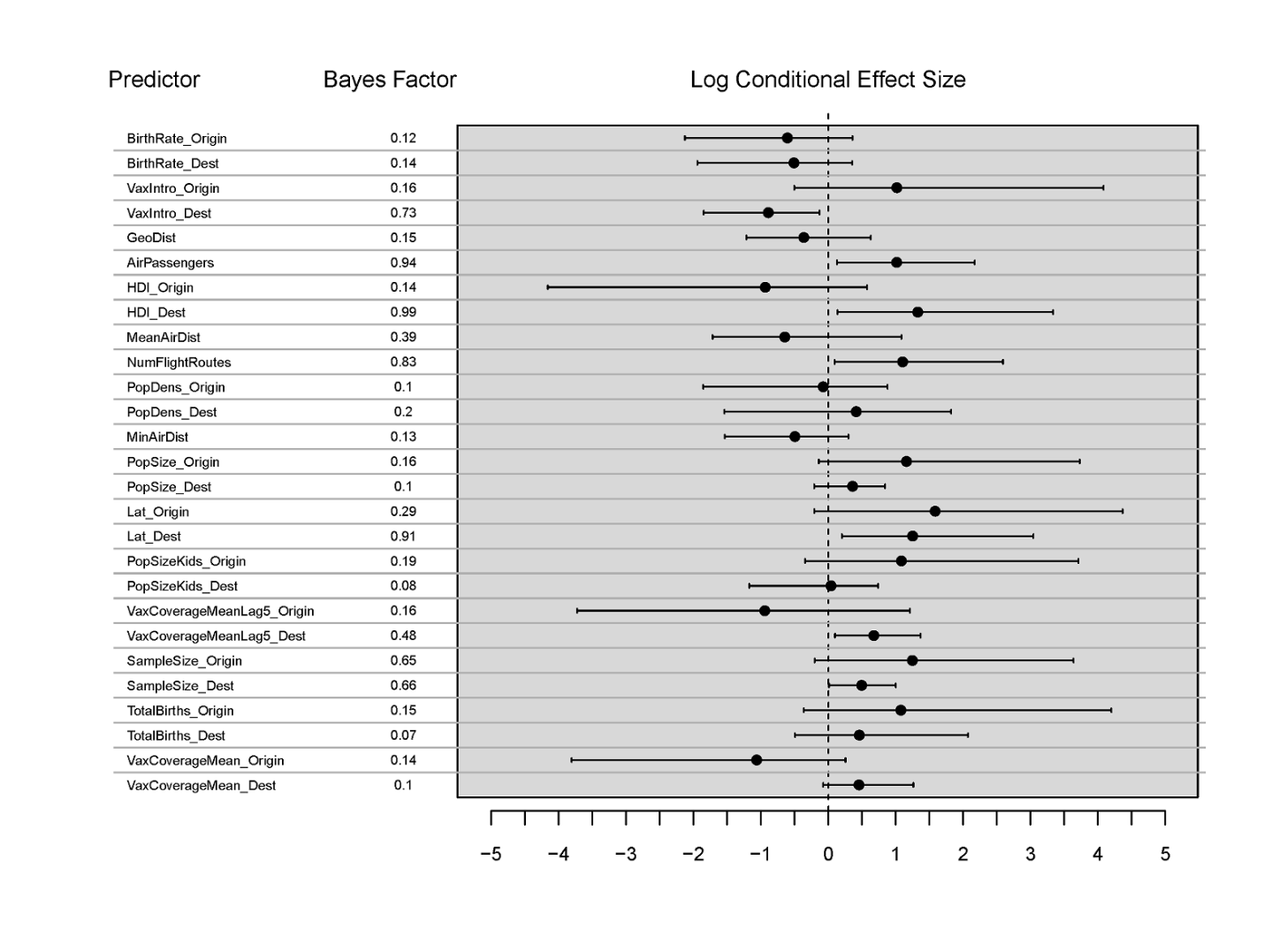


**Fig. S7. Bayesian phylogenetic GLM analysis of E/SE Asia sequences for genotype 1E from the most recent epidemics (2011-2015).** No predictors were significant.


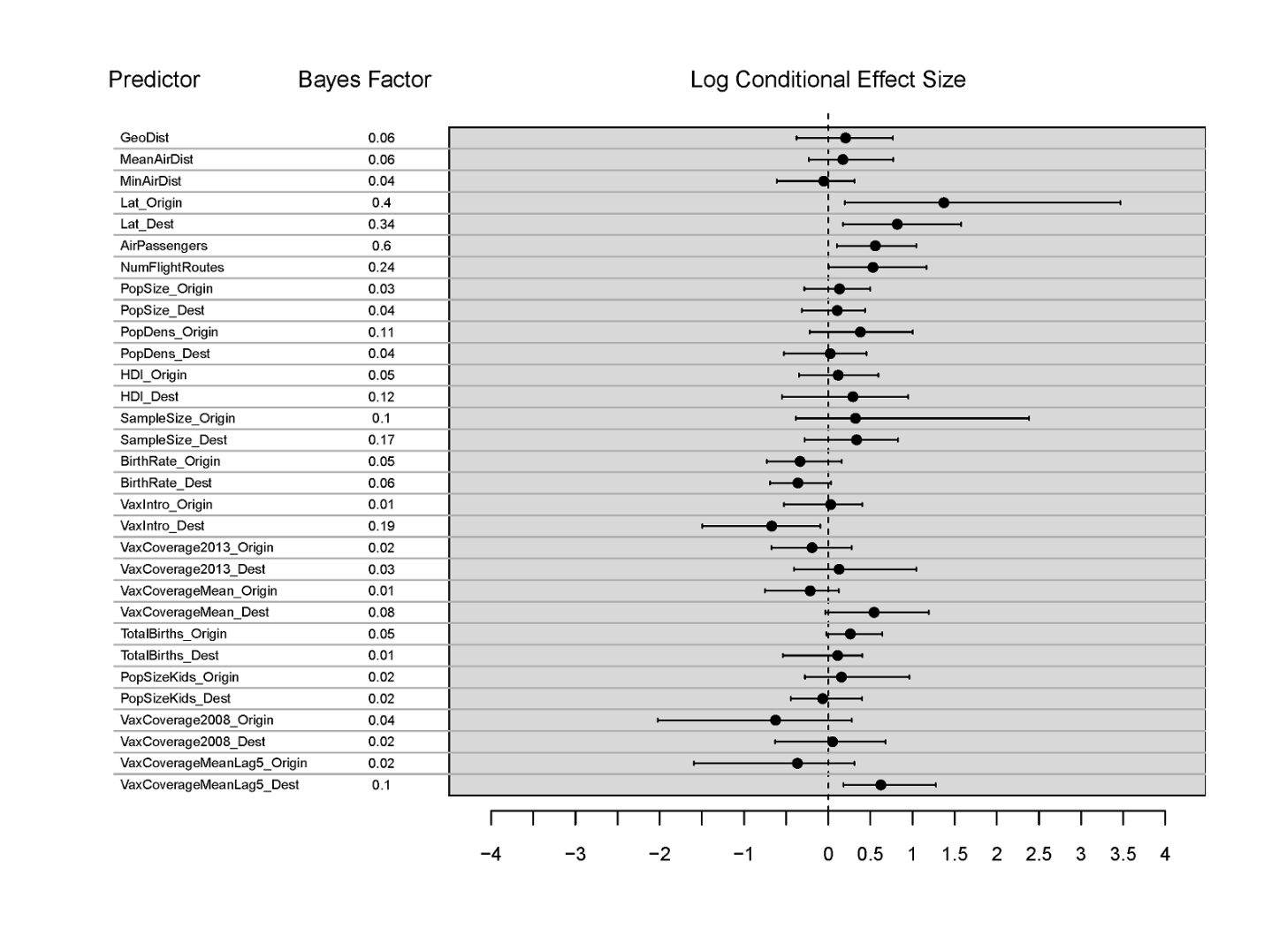


**Fig. S8. Bayesian phylogenetic GLM analysis of all E/SE Asia sequences for genotype 2B (2000-2016).** No predictors were significant


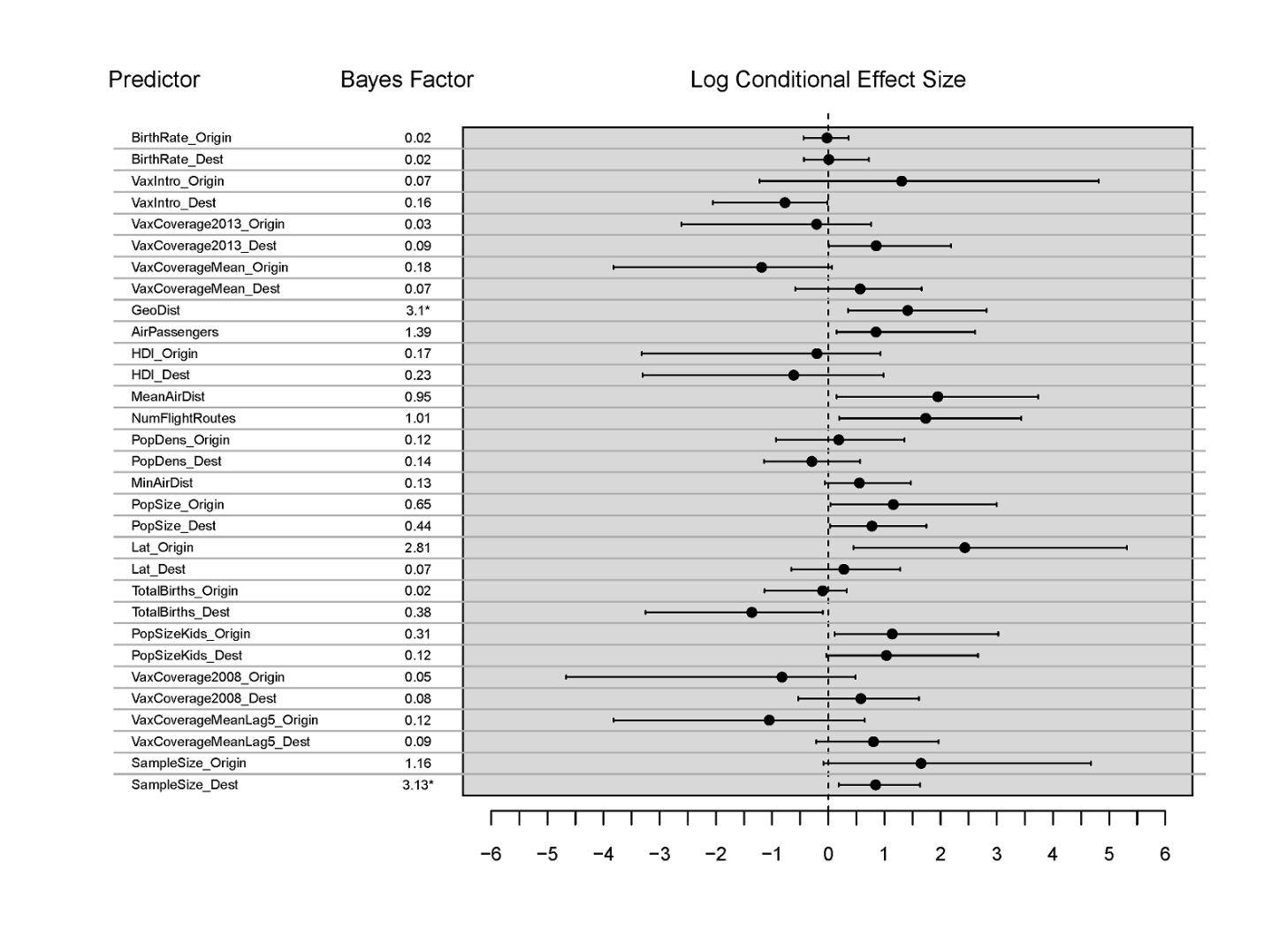


**Fig. S9. Bayesian phylogenetic GLM analysis of E/SE Asia sequences for genotype 2B from the most recent epidemics (2011-2015).** An asterisk follows Bayes factors for significant predictors.


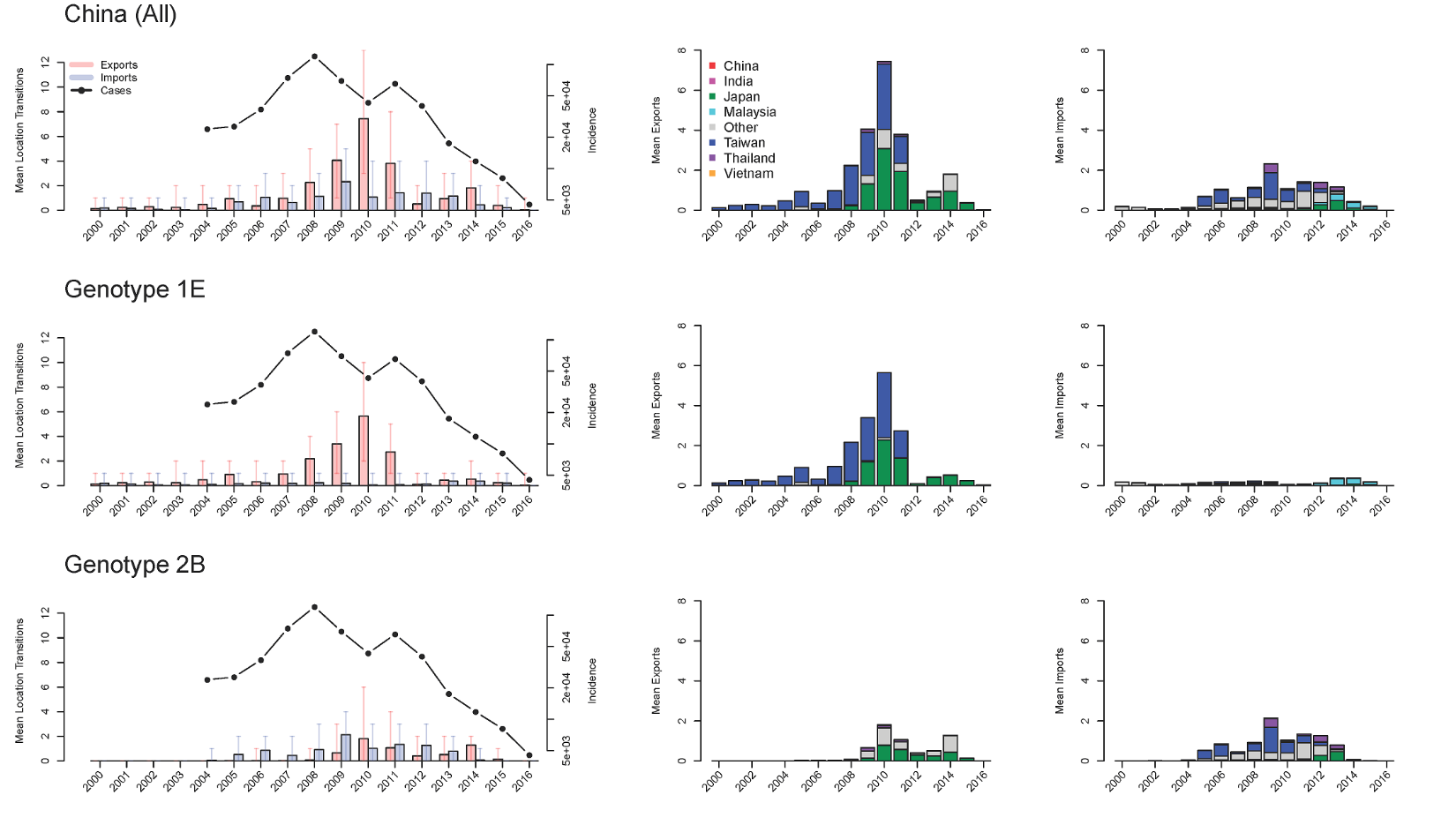


**Fig. S10.** **China viral import/export profile.** WHO incidence superimposed onto a bar plot of the mean number of viral transitions into (blue) and out of (red) China by year (left column). Destination of exports (center column) and origin of imports (right column) of all (top row), genotype 1E (middle row) and genotype 2B (bottom row) viral transitions out of and into China by year.


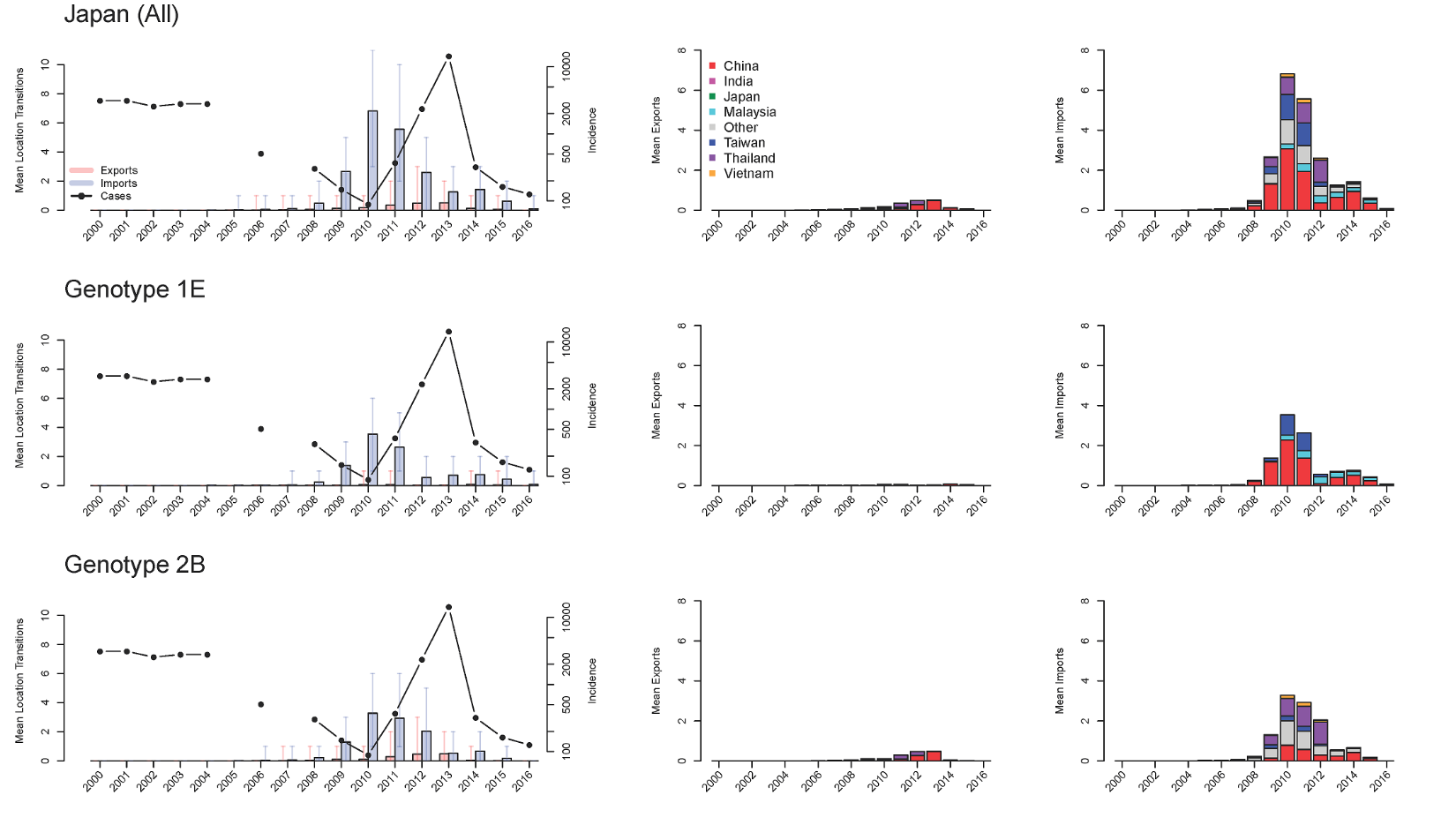


**Fig. S11.** **Japan viral import/export profile.** WHO incidence superimposed onto a bar plot of the mean number of viral transitions into (blue) and out of (red) Japan by year (left column). Destination of exports (center column) and origin of imports (right column) of all (top row), genotype 1E (middle row) and genotype 2B (bottom row) viral transitions out of and into Japan by year.


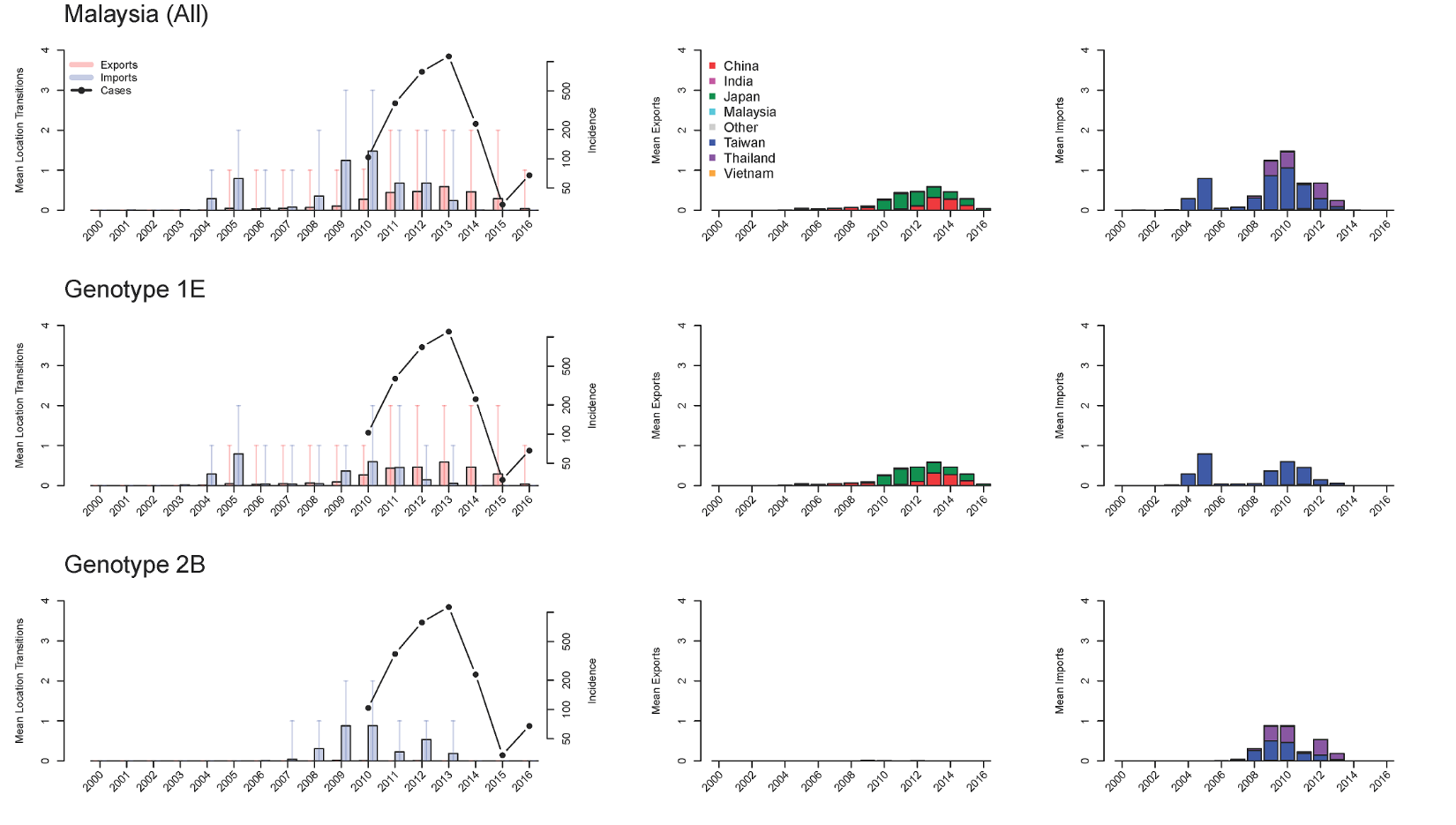


**Fig. S12.** **Malaysia viral import/export profile.** WHO incidence superimposed onto a bar plot of the mean number of viral transitions into (blue) and out of (red) Malaysia by year (left column). Destination of exports (center column) and origin of imports (right column) of all (top row), genotype 1E (middle row) and genotype 2B (bottom row) viral transitions out of and into Malaysia by year.


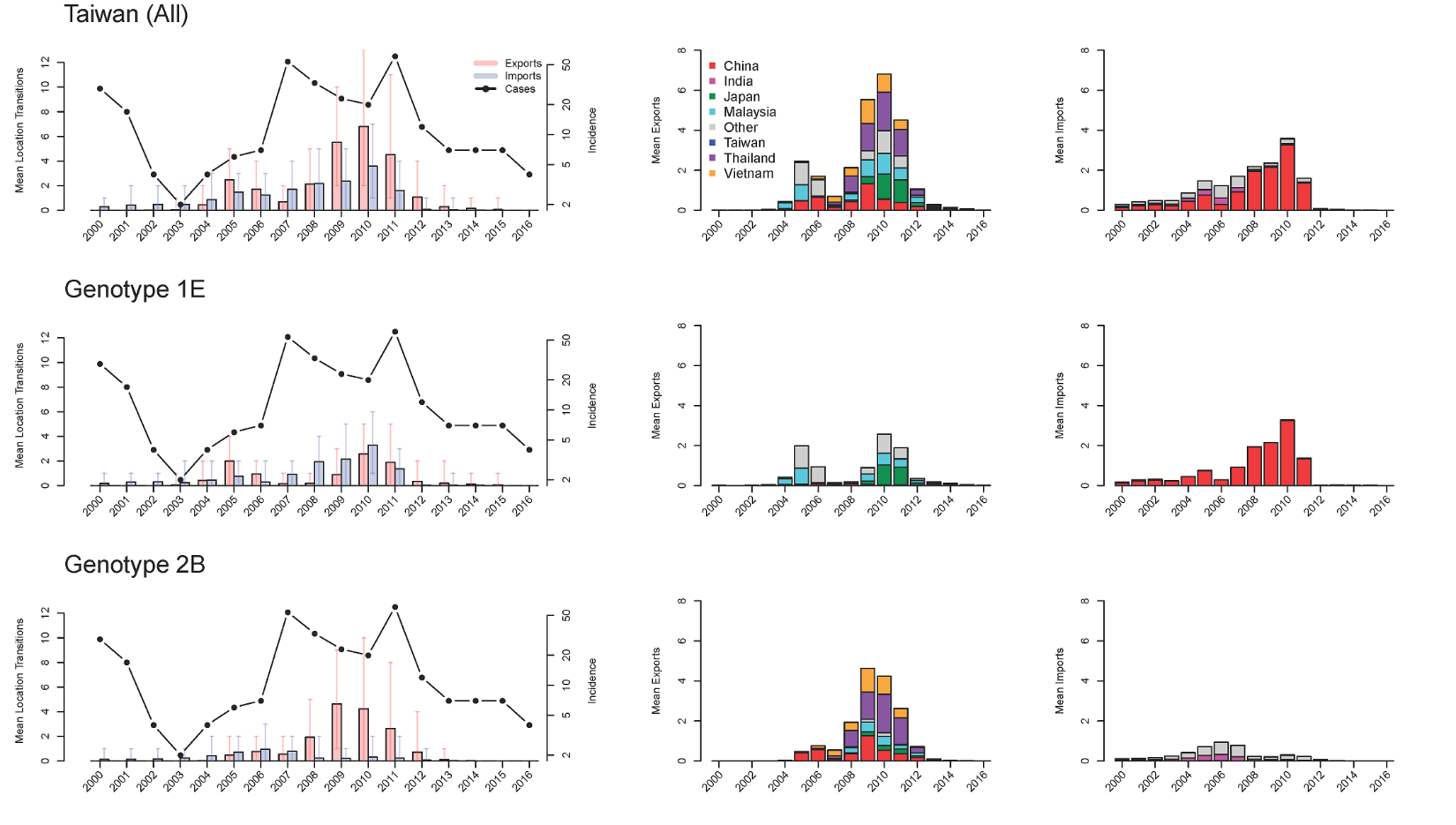


**Fig. S13.** **Taiwan viral import/export profile.** WHO incidence superimposed onto a bar plot of the mean number of viral transitions into (blue) and out of (red) Taiwan by year (left column). Destination of exports (center column) and origin of imports (right column) of all (top row), genotype 1E (middle row) and genotype 2B (bottom row) viral transitions out of and into Taiwan by year.


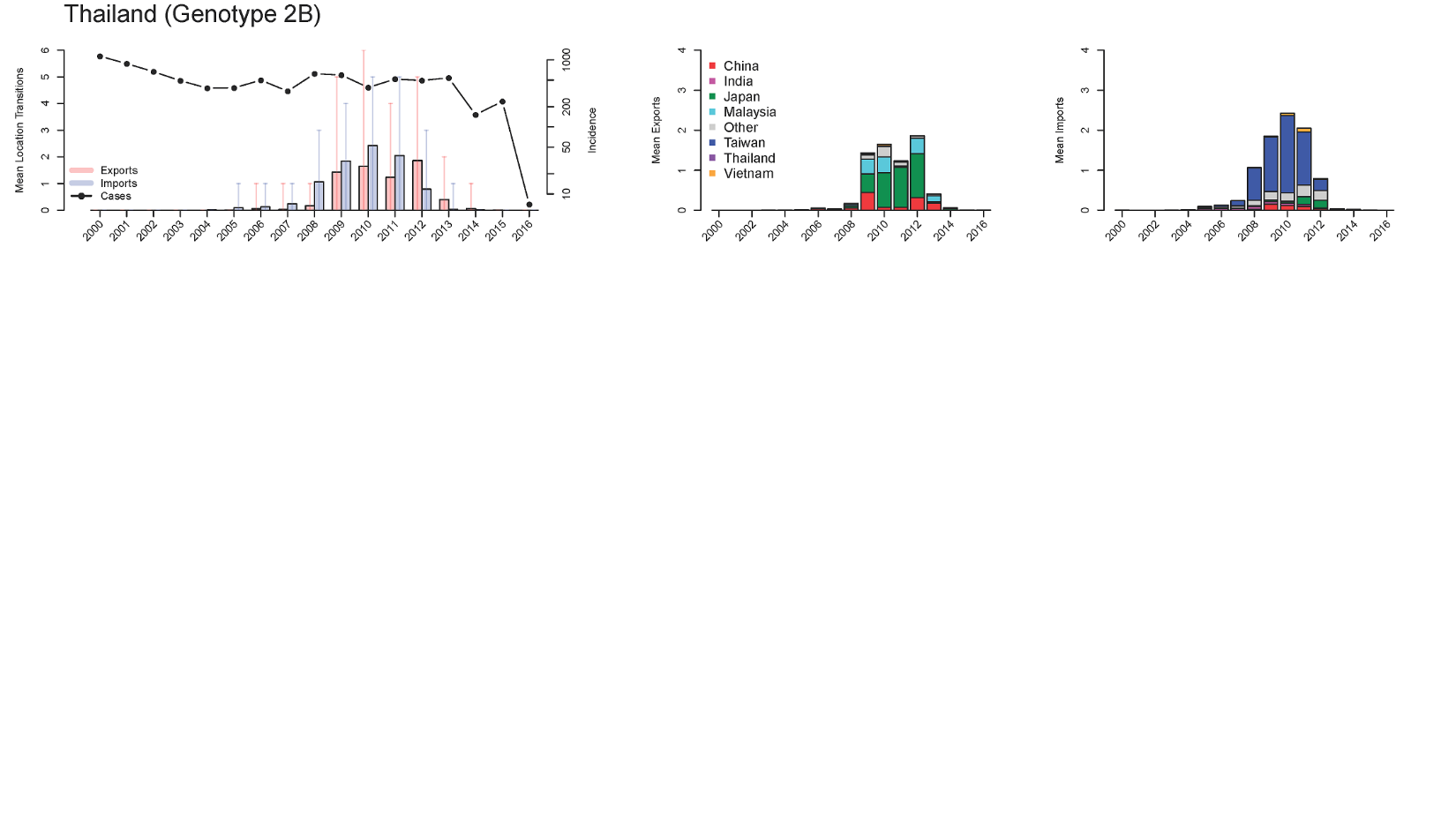


**Fig. S14.** **Thailand viral import/export profile.** WHO incidence superimposed onto a bar plot of the mean number of viral transitions into (blue) and out of (red) Thailand by year (left). Destination of exports (center) and origin of imports (right) of viral transitions out of and into Thailand by year. As of 2016, only genotype 2B viruses have been recovered in Thailand.


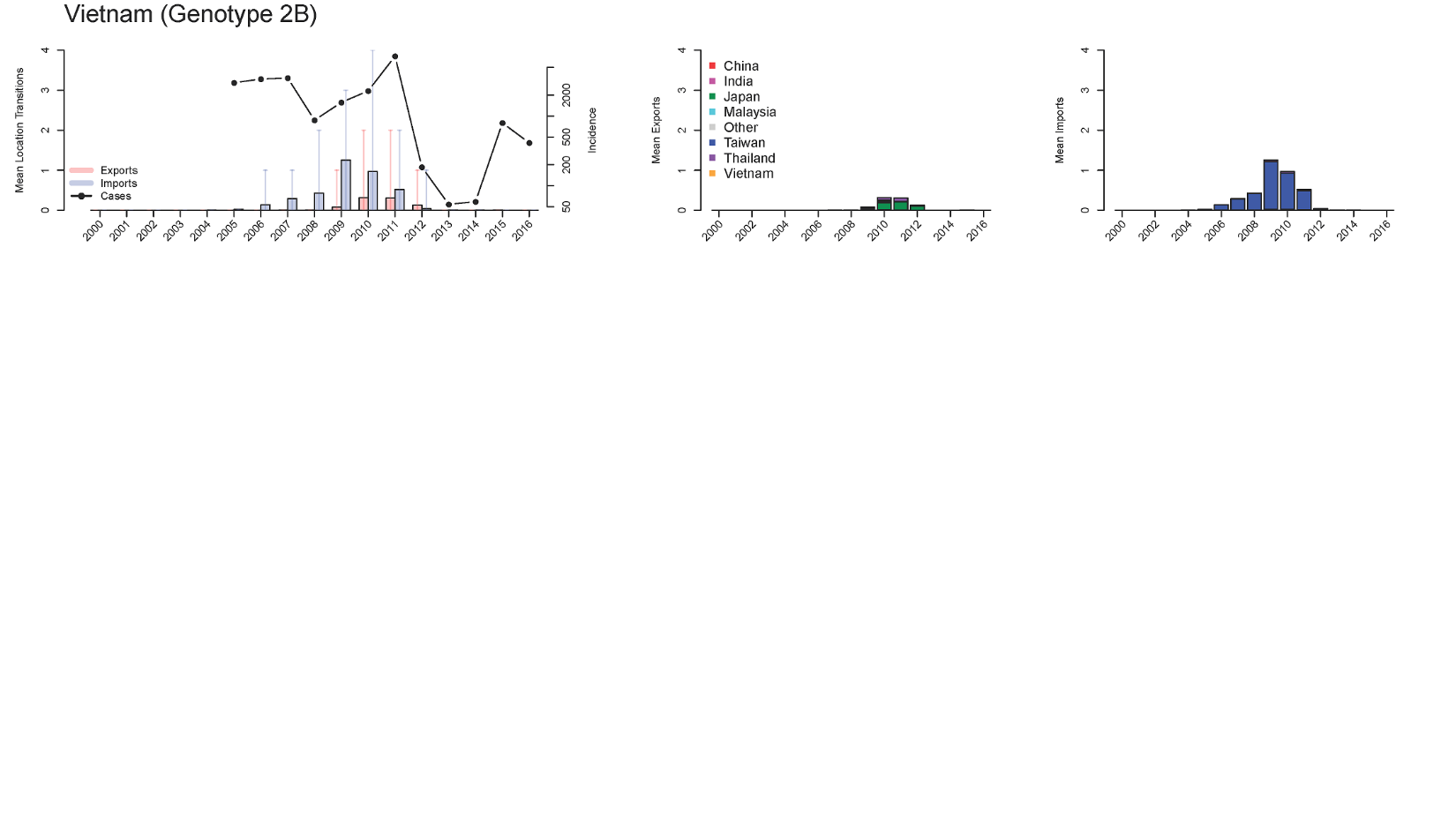


**Fig. S15.** **Vietnam viral import/export profile.** WHO incidence superimposed onto a bar plot of the mean number of viral transitions into (blue) and out of (red) Vietnam by year (left). Destination of exports (center) and origin of imports (right) of viral transitions out of and into Vietnam by year. As of 2016, only genotype 2B viruses have been recovered in Vietnam.

**
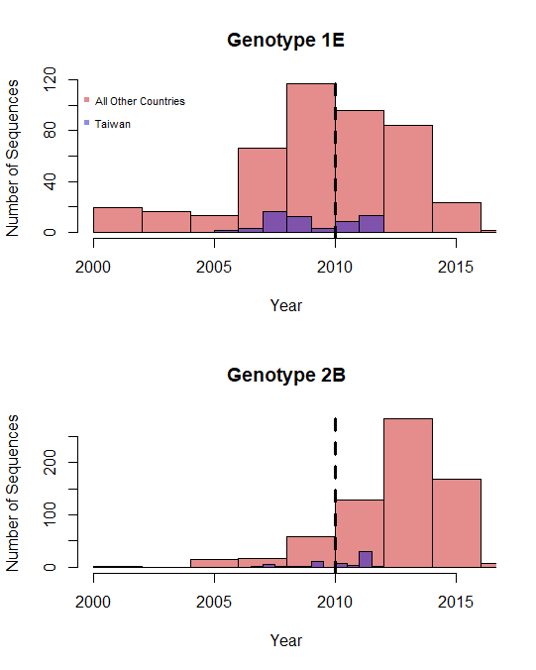
**

**Fig. S16. Histogram of sequences collected from Taiwan (purple) and all other countries (pink) by year.** Genotype 1E (top) and genotype 2B (bottom).

**
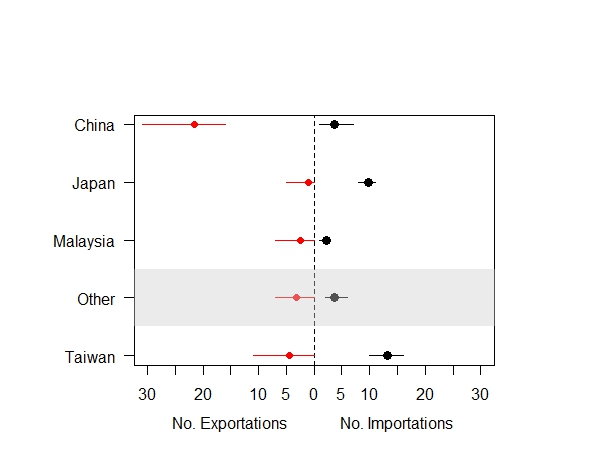

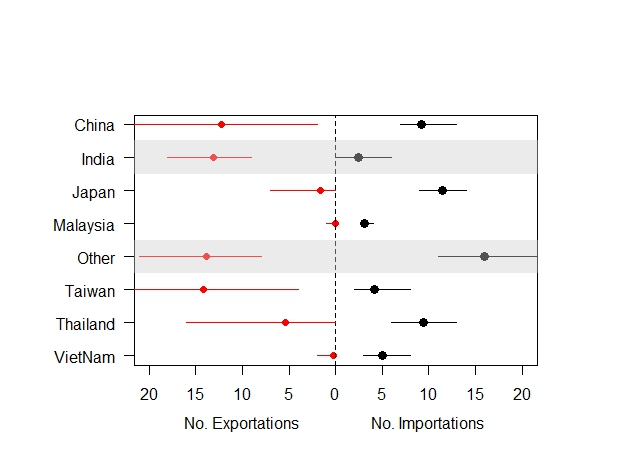
**
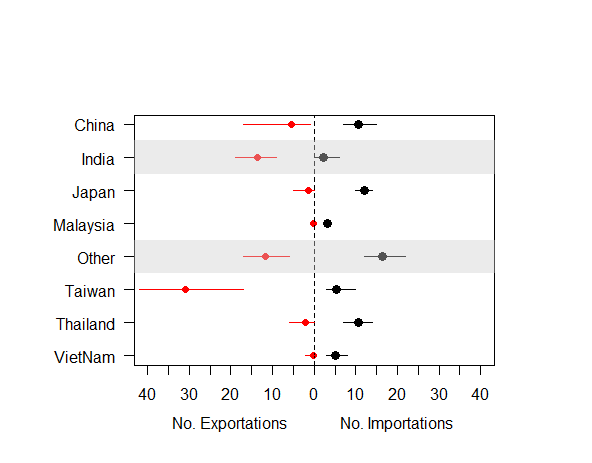


**Fig S17. Imports and exports by country, Taiwan sequences collected prior to 2010 removed.** left) genotype 1E; center) genotype 2B, subsampled set of 24 Taiwan sequences; right) genotype 2B, full dataset of 41 Taiwan sequences


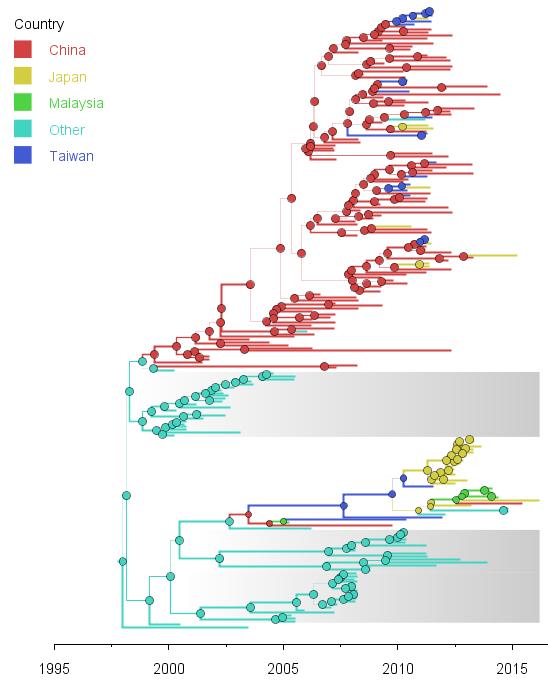


**Fig S18. Genotype 1E phylogeny with Taiwan sequences collected prior to 2010 removed.** Branch colors represent the most probable location state of the lineage at that time. X-axis represented in years. Branch widths sized according to posterior probability, where thicker branches indicate greater posterior support. Clades shaded in grey represent continuous transmission of a lineage outside of Asia. Interior nodes are colored based on their inferred geographic location and sized by the posterior support for this placement, where larger circles indicate greater posterior support.


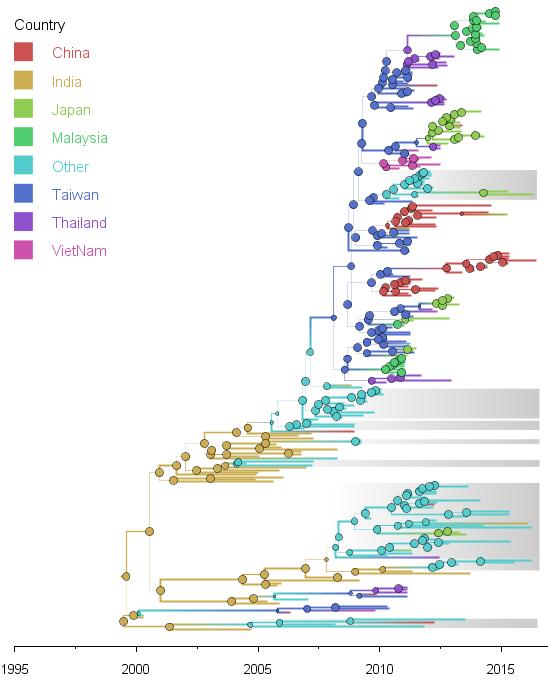


**Fig S19. Genotype 2B phylogeny, full dataset of 41 Taiwan sequences collected post-2010 (inclusive).** Branch colors represent the most probable location state of the lineage at that time. X-axis represented in years. Branch widths sized according to posterior probability, where thicker branches indicate greater posterior support. Clades shaded in grey represent continuous transmission of a lineage outside of Asia. Interior nodes are colored based on their inferred geographic location and sized by the posterior support for this placement, where larger circles indicate greater posterior support.

**
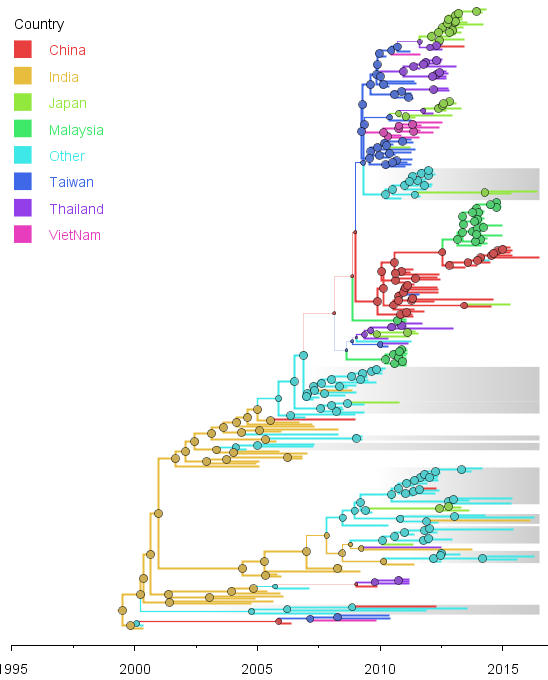

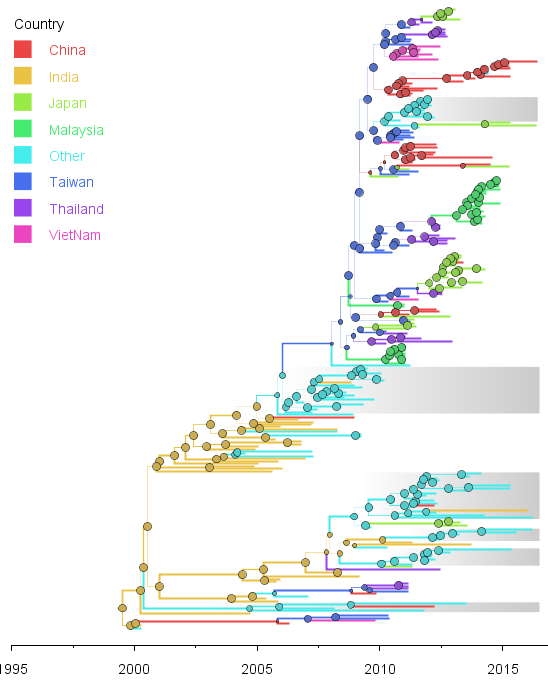

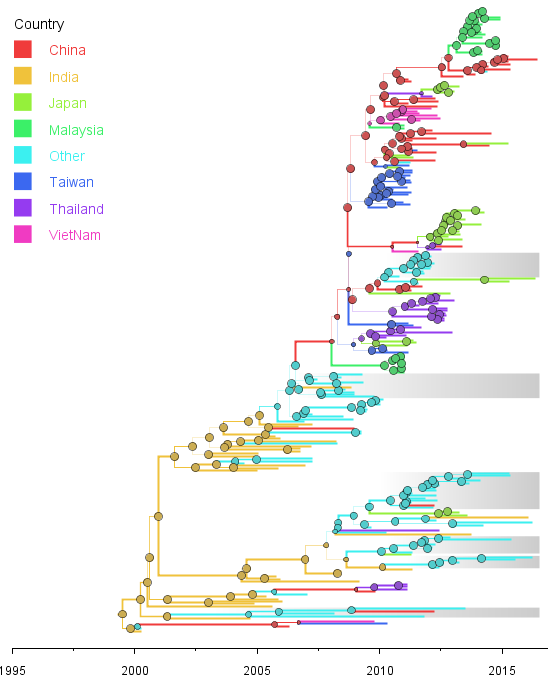
**

**Fig S20. Genotype 2B phylogenies of 3 subsampled sets of 24 Taiwan sequences collected post-2010 (inclusive).** Branch colors represent the most probable location state of the lineage at that time. X-axis represented in years. Branch widths sized according to posterior probability, where thicker branches indicate greater posterior support. Clades shaded in grey represent continuous transmission of a lineage outside of Asia. Interior nodes are colored based on their inferred geographic location and sized by the posterior support for this placement, where larger circles indicate greater posterior support.
